# Influence of late Pleistocene sea-level variations on midocean ridge spacing in faulting simulations and a global analysis of bathymetry

**DOI:** 10.1073/pnas.2204761119

**Published:** 2022-07-07

**Authors:** Peter Huybers, Parker Liautaud, Cristian Proistosescu, Bridgit Boulahanis, Suzanne M. Carbotte, Richard F. Katz, Charles Langmuir

**Affiliations:** ^a^Department of Earth and Planetary Sciences, Harvard University, Cambridge, MA 02138;; ^b^Department of Atmospheric Sciences, University of Illinois at Urbana–Champaign, Urbana, IL 61801;; ^c^Department of Geology, University of Illinois at Urbana–Champaign, Urbana, IL 61801;; ^d^Lamont-Doherty Earth Observatory, Columbia University, New York, NY 10034;; ^e^Department of Earth Sciences, University of Oxford, Oxford OX1 3AN, United Kingdom

**Keywords:** volcanism, midocean ridges, sea level, Milankovitch forcing

## Abstract

It is established that changes in sea level influence melt production at midocean ridges, but whether changes in melt production influence the pattern of bathymetry flanking midocean ridges has been debated on both theoretical and empirical grounds. To explore the dynamics that may give rise to a sea-level influence on bathymetry, we simulate abyssal hills using a faulting model with periodic variations in melt supply. For 100-ky melt-supply cycles, model results show that faults initiate during periods of amagmatic spreading at half-rates >2.3 cm/y and for 41-ky melt-supply cycles at half-rates >3.8 cm/y. Analysis of bathymetry across 17 midocean ridge regions shows characteristic wavelengths that closely align with the predictions from the faulting model. At intermediate-spreading ridges (half-rates >2.3 cm/y and ≤3.8 cm/y) abyssal hill spacing increases with spreading rate at 0.99 km/(cm/y) or 99 ky (*n* = 12; 95% CI, 87 to 110 ky), and at fast-spreading ridges (half-rates >3.8 cm/y) spacing increases at 38 ky (*n* = 5; 95% CI, 29 to 47 ky). Including previously published analyses of abyssal-hill spacing gives a more precise alignment with the primary periods of Pleistocene sea-level variability. Furthermore, analysis of bathymetry from fast-spreading ridges shows a highly statistically significant spectral peak (*P* < 0.01) at the 1/(41-ky) period of Earth’s variations in axial tilt. Faulting models and observations both support a linkage between glacially induced sea-level change and the fabric of the sea floor over the late Pleistocene.

## Significance

We address the hypothesis that changes in Pleistocene sea level have consequences for abyssal-hill bathymetry. A model illustrates how reductions in magma production caused by rising sea level could trigger faults at 100-ky intervals at intermediate spreading-rate ridges (>2.3 cm/y) and at 41-ky intervals at faster spreading-rate ridges (>3.8 cm/y). Analysis of 17 different regional ridge systems gives characteristic length scales that closely align with the predictions from the faulting model. Furthermore, a robust spectral peak is found at the 41-ky obliquity period at fast-spreading ridges. Together, these results constitute strong evidence for a pervasive influence of Pleistocene variations in glaciation and sea level on the pattern of abyssal hills.

Parallel to the global system of midocean ridges is an orderly progression of fault-bounded topographic highs called abyssal hills. Hypothesized models for abyssal-hill formation generally involve magma delivery rates, either directly through variable magma emplacement at the surface (1) or indirectly by modulating the amount of plate extension that must be accommodated by faulting (2–5). Faults would be especially likely to occur during times of reduced magma supply, when stress accumulates as young lithosphere is pulled away from a spreading axis (6, 7).

Abyssal hills exhibit regularity in spacing and symmetry across the ridge axis (1) that has motivated hypotheses of periodic or quasiperiodic variability in magma supply (4, 6–10), but the causes of such variability have been obscure. One proposed mechanism involves sea-level change driven by glacial cycles (11–13). Melting beneath ridges is driven by depressurization of upwelling mantle and hence the melting rate can be modulated by pressure variations associated with fluctuating sea level. During the last deglaciation, for example, sea level rose by ∼1 cm/y for 10,000 y, suggesting an ∼10% reduction in rates of melt generation for mantle upwelling at 3 cm/y, where the factor of 3 difference in density between mantle and seawater is taken into account. More sophisticated models that account for melt migration also predict sea-level–induced variations in melt supply beneath ridges (14) and that such variations will depend upon factors including spreading rate and mantle permeability (12).

Throughout the late Pleistocene, variations in sea level occur most prominently at 18- to 23-, 41-, and 100-ky periods (15). The 18- to 23- and 41-ky periods are, respectively, associated with variations in the orientation and tilt of Earth’s spin axis, whereas the origin of the 100-ky variability appears to involve a combination of orbital variations, glacial dynamics, and variations in atmospheric CO_2_ (16). Although the 100-ky cycle dominates the magnitude of sea-level variability during the late Pleistocene, melt-supply variations respond to the rate of change of sea level, accentuating the importance of higher-frequency variations (*SI Appendix*, Fig. S1) (12, 13).

The hypothesis that Pleistocene variations in sea level influence melt supply is supported by several lines of evidence. Proxies of ridge hydrothermal circulation indicate a response to sea-level change (17–19), as do variations in the abundance of sediment-hosted volcanoclastics (20). Axis-parallel variations in crustal thickness along the East Pacific Rise of 200 to 800 m vary with wavelengths that are consistent with the sea-level hypotheses (21). Bathymetry along portions of the Australian–Antarctic ridge (12) and Chile Rise (22) was found to contain significant concentrations of variability at orbital and 100-ky periods. Finally, an analysis of characteristic spacing involving five regions whose half-spreading rate exceeds 3 cm/y found that the spacing across the fastest-spreading regions appeared to increase with spreading rate, consistent with the possibility that orbital cycles influence spacing (23).

It has been argued, however, that sea-level–induced variations in melt supply would have negligible implications for surface bathymetry on two counts. First, the presence of a magma chamber at the ridge axis could damp the influence of melt supply, and, second, flexural rigidity limits surface bathymetry variations associated with emplacement of magma at the base of the crust (24). Furthermore, there is a long-standing interpretation that the regularity in midocean ridge bathymetry reflects preferred length scales inherent to lithospheric structural controls (3, 25, 26), such that the presence of quasiperiodicity in bathymetry is not itself indicative of a response to orbital or 100-ky variations in climate (24).

**Fig. 1.**
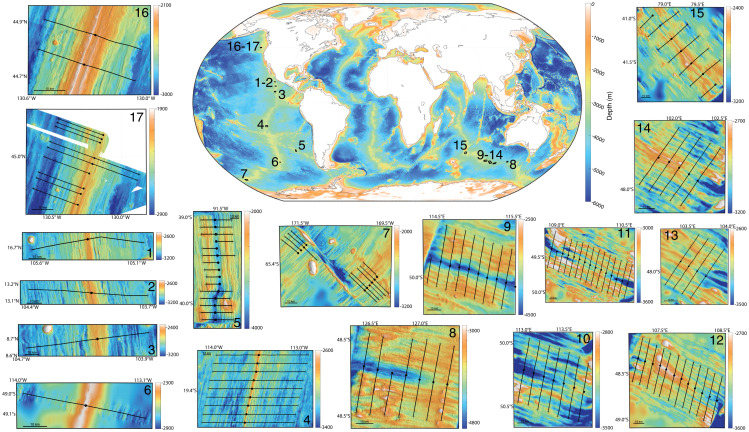
Global bathymetry with details of the 17 abyssal-hill regions used in this study. Regional maps show ridge flank profiles along which center-beam bathymetry is extracted (black lines) that extend from the ridge axis (circles) to the estimated location of the Brunhes–Matuyama magnetic reversal (crosses). A total of 182 ridge flank profiles adjoin to give 91 complete transects of the ridge, whereas 23 ridge flank profiles are unpaired because the other side is disturbed, the Brunhes–Matuyama reversal is not identifiable, or bathymetry data are missing. Mapped bathymetry is from the Marine Geoscience Data System. White shading indicates that data are unavailable. Regional maps show a 10-km horizontal scale (horizontal black bar).

We seek to distinguish between abyssal-hill length scales that are inherent to lithospheric structural controls and those that reflect temporal periodicity in sea level and magma supply, such as at orbital periods. Our primary approach is to examine differing predictions for the relationship between spreading rate and abyssal-hill spacing at intermediate and faster spreading ridges. An assumption that melt supply accommodates a constant fraction of spreading (3) leads to a conclusion that abyssal-hill spacing becomes constant at half-spreading rates above ∼3 cm/y (e.g., ref. 24, their figure 3a and supplemental material). Conversely, if abyssal-hill spacing is controlled by quasiperiodic variability in melt, characteristic wavelengths should increase with spreading rate in proportion to the period of melt variability (23). To explore these distinct predictions, we first revisit a faulting model that was used to illustrate how quasiperiodic bathymetry can be independent of sea-level variations (24) to explore conditions under which sea-level–induced changes in melt supply influence simulated bathymetry. We then compare our simulations against analyses of bathymetric variability across 17 different regions (Fig. 1 and Table 1).

**Table 1.** Data sources for each region from which bathymetry is analyzed in this study

Region no.	Cruise designation	Year	Principal investigator(s)
1	PANR01MV	1997	C. Langmuir
2	PPTU01WT	1985	J. McClain
3	EW9708	1997	D. Toomey
4	SOJN01MV	1997	D. Scheirer
5	PANR04MV	1998	J. Karsten, E. Klein
6	NBP9707	1997	S. Cande, J. Stock
7	EW9201	1992	S. Cande et al.
8	BMRG05MV	1996	J. Sempere, D. Christie
9	WEST09MVa	1994	J. Cochran, J. Sempere
10	WEST09MVb	1994	J. Cochran, J. Sempere
11	WEST09MVc	1994	J. Cochran, J. Sempere
12	WEST09MVd	1994	J. Cochran, J. Sempere
13	WEST09MVe	1994	J. Cochran, J. Sempere
14	WEST09MVf	1994	J. Cochran, J. Sempere
15	BMRG06MV	1996	K. Johnson
16	WEST15MV	1995	S. Cande, J. Hildebrand
17	AT26-19	2015	C. Langmuir, S. Carbotte

Regions 1 to 4, 6, and 7 trend north to south along the East Pacific Rise; region 5 is from the Chile Ridge; regions 8 to 15 trend east to west along the Southeast Indian Ridge; and regions 16 and 17 are from the Juan de Fuca Ridge.

## 1. Faulting Simulations Using Sea-Level–Driven Variations in Magma Supply

We use the fast Lagrangian analysis of continua (FLAC) model (3, 4), to explore how magma-supply variations produced by sea-level change could influence faulting and abyssal-hill spacing. FLAC represents stress, strain, and faulting in a vertical plane that transects a midocean ridge perpendicular to its strike (*SI Appendix*, Fig. S2), and it has been widely used in studies of midocean ridge faulting (2, 5). An earlier study (24) used FLAC to describe abyssal hills as fault-bounded blocks that result from plate separation being incompletely accommodated by magmatic emplacement. They argued that midocean ridge faults have an intrinsic spacing that is generally insensitive to variations in magma supply at 100-ky and shorter periods. To facilitate comparison we use the same FLAC parameter values as those in a previous study (24) in our main line of analysis (Table 2), changing only the period of melt-supply variations and spreading rates.

**Table 2.** Default parameters used in FLAC runs in the main text

Parameter	Value	Description
*U*_0_	1 to 10 cm/y	Spreading half-rate
*M*	0.85	Fraction of magmatic accommodation
*τ*	41 or 100 ky	Forcing period
$\eta_{\text{inj}}$	10^19^ Pa · s	Maximum injection-zone viscosity
$\Delta x_{\text{inj}}$	2 km	Width of magmatic injection zone
$\tau_{\text{heal}}$	$5\times 10^{12}$ s	Healing parameter
*L*	$5\times 10^{5}$ J/kg	Latent heat
$T_{\text{sfc}}$	0 ${}^{{}^{\circ}}$C	Surface temperature
*n*	6 and 12	Nusselt numbers for lower and upper layers
*z_n_*	7 km	Maximum depth for hydrothermal effects
*T_n_*	400 ${}^{{}^{\circ}}$C	Maximum temperature for hydrothermal effects

Maximum injection-zone viscosity is specific to the model formulation of ref. 4.

**Fig. 2.**
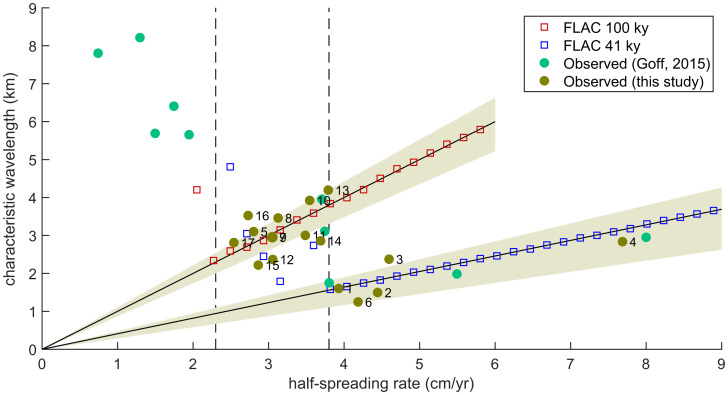
Scaling of abyssal-hill spacing with spreading rate produced by a numerical faulting model (FLAC) driven by magmatic cycles having 100-ky (red squares) or 41-ky period (blue squares). At slow-spreading rates (≤2.3 cm/y) simulated fault spacing is inversely proportional to spreading rates, but at half-spreading rates >2.3 cm/y (left vertical dashed line) faults lock into 100-ky spacing (steeper solid black line) and at half-spreading rates >3.8 cm/y (right vertical dashed line) faults lock into 41-ky spacing (shallower solid black line). Observationally derived estimates of characteristic abyssal-hill spacing are shown (brown circles) for the 17 regions analyzed in this study (Fig. 1), as well as abyssal-hill spacing estimates reported in a previous study (23) (green circles). Across intermediate-spreading regions (5, 7–17) characteristic spacing has a slope of 0.99 km ⋅cm^–1^ ⋅y^–1^ or 99 ky (95% CI 87 to 110 ky, upper beige shading), and fast-spreading regions (1–4, 6) have a slope of 38 ky (95% CI 29 to 47 ky, lower beige shading).

The spacing of abyssal hills in FLAC simulations is a function of spreading rate, melt supply, and the evolving strength of the lithosphere. Fig. 2 shows the results of FLAC simulations with half-spreading rates ranging between 2 and 9 cm/y and melt cycles that alternate between magmatic and amagmatic phases over 41- or 100-ky cycles. These melt-cycle periods correspond with the two largest-amplitude periods of sea-level variability during the late Pleistocene and are expected to cause changes in magma supply (11–14).

At slow spreading rates FLAC produces abyssal-hill spacing that decreases with increasing spreading rate (3), consistent with observations (25). This relationship can be understood by the forces required to reactivate slip on a fault versus initiating a new fault in the thinner brittle layer nearer the axis (3, 4). The pulling force required to reactivate a fault generally increases over time because of thickening of the lithosphere as the fault is rafted away from the spreading axis. When inactive, the fault also recovers cohesion lost to strain weakening. If the force required to reactivate a fault exceeds the strength of the unfaulted lithosphere, the existing fault is abandoned and a new fault is initiated closer to the spreading axis. At slow spreading rates the unfaulted lithosphere is thick and sufficiently strong relative to an existing fault that multiple melt cycles elapse before initiation of a new fault. Increases in spreading rate at slow spreading rates cause existing faults to be abandoned sooner and abyssal hills to be more closely spaced because such increases in spreading rate lead to a thinner axial lithosphere.

Above a certain spreading-rate threshold, however, a new fault initiates during each amagmatic phase, such that the spacing between abyssal hills follows either 100 or 41 ky times the half-spreading rate (Fig. 2). This regime in which magma-supply period controls abyssal-hill spacing was shown in earlier studies using FLAC (4, 24) but using a smaller number of simulations and by varying the period of the magma cycle, instead of the spreading rate. We find that the spreading-rate threshold for 41-ky melt cycles is 3.8 cm/y (half rate), a value that corresponds with an empirically identified change in the relationship between spreading rate and abyssal-hill spacing (23). The spreading-rate threshold for 100-ky melt cycles is 2.3 cm/y. We infer that a new fault initiating every amagmatic interval occurs at a slower spreading rate for 100- than 41-ky melt cycles because the longer cycle gives more time for the fault to strengthen.

It should be noted, following previous studies (4, 24), that plate spreading in FLAC is alternately accommodated entirely by magma supply or entirely by faulting during amagmatic phases. This representation is an upper bound on magma-supply variability and is substantially larger than the order 10% variations predicted by foregoing models of melt generation (12, 14). These models, however, do not account for phenomena associated with melt-channelization dynamics that could lead to greater-amplitude variations in melt supply (27, 28). Although the FLAC model is inevitably incomplete, it nonetheless gives specific predictions regarding the relationship between spreading rate and fault spacing that can be compared to observations.

## 2. Observational Analyses of Late-Pleistocene Bathymetry

We evaluate abyssal-hill spacing at intermediate- and faster-spreading ridges. Relevant observations come from multibeam swath bathymetry from transects that span the late Pleistocene. We aim for global ocean-ridge coverage (Fig. 1), although there are limitations in the Atlantic and portions of the East Pacific. The mid-Atlantic ridge is not included because its spreading rates are generally below the FLAC-response threshold of 2.3 cm/y and because a sluggish magmatic ascent is expected to attenuate sea-level–driven fluctuations in magma supply (12). Many ridge flank profiles from the East Pacific Rise are also unusable because of the abundance of off-axis volcanism. In total, 205 bathymetry ridge flank profiles are included from 17 regions whose average spreading rates exceed 2.3 cm/y. Regions include the East Pacific Rise, Southeast Indian Ridge, Chile Rise, and Juan de Fuca ridges. As described in more detail in *Materials and Methods*, age models are developed for each profile by linearly interpolating age with distance between the ridge axis and the Brunhes–Matuyama magnetic reversal, assigned an age of 780 ka (29).

### A. Characteristic Abyssal-Hill Spacing.

We obtain a characteristic wavelength for the abyssal hills in each region through identifying the maximum spectral density in the gradient of bathymetry (*SI Appendix*, Fig. S4). This characteristic wavelength is indicative of the corner wavelength in a Matérn process, an established stochastic description of seafloor bathymetry variability (25), as well as a concentration of spectral energy superimposed on a background continuum, as elsewhere posited (12, 22) (*Materials and Methods*). Analyses are divided into two groups corresponding to intermediate (>2.3 cm/y and ≤3.8 cm/y) and fast (>3.8 cm/y) half-spreading rates, as predicted by the FLAC simulations.

The critical test is whether abyssal-hill spacing increases with spreading rate at the glacial periods of 41 and 100 ky. Indeed, regressing characteristic spacing against spreading rates across intermediate-spreading regions gives a slope of 0.99 km ⋅cm^–1^ ⋅y^–1^ (95% CI, 0.87 to 1.10 km ⋅cm^–1^ ⋅y^–1^). Simplifying the units, the slope is 99 ky or almost precisely the scaling predicted by FLAC in response to 100-ky melt cycles. Fast-spreading regions give a slope of 38 ky (95% CI, 29 to 47 ky) that aligns closely with hill spacing controlled by 41-ky obliquity cycles. Previously published abyssal-hill wavelengths (23) also align well with our FLAC results. Inclusion of these additional data points narrows the regression CIs for both the intermediate- (95% CI, 88 to 108 ky) and fast-spreading ridges (95% CI, 33 to 43 ky) to values that even more precisely encompass the FLAC predictions.

### B. Spectral Analysis of Bathymetry.

A complementary analysis is possible in the frequency domain, for which we use a multitaper spectral estimation procedure on individual ridge flank bathymetry profiles. The presence of a strongly red background spectrum makes it useful to prewhiten spectral estimates before testing for the presence of spectral peaks (30). A concern, however, is whether it is possible to distinguish an inflection point in the spectrum—as found, for example, in a Matérn process—from a spectral peak after prewhitening (10). Synthetic tests indicate that prewhitening the spectrum of a Matérn process can lead to the appearance of a spectral peak at 1/(100 ky) because this peak is among the lowest frequencies resolved in a 780-ka-long record (*SI Appendix*, Fig. S5). At the 1/(41-ky) frequency, however, inflections and peaks are readily distinguished (*SI Appendix*, Fig. S5), leading us to focus on the higher-frequency fluctuations found at faster-spreading rates.

**Fig. 3.**
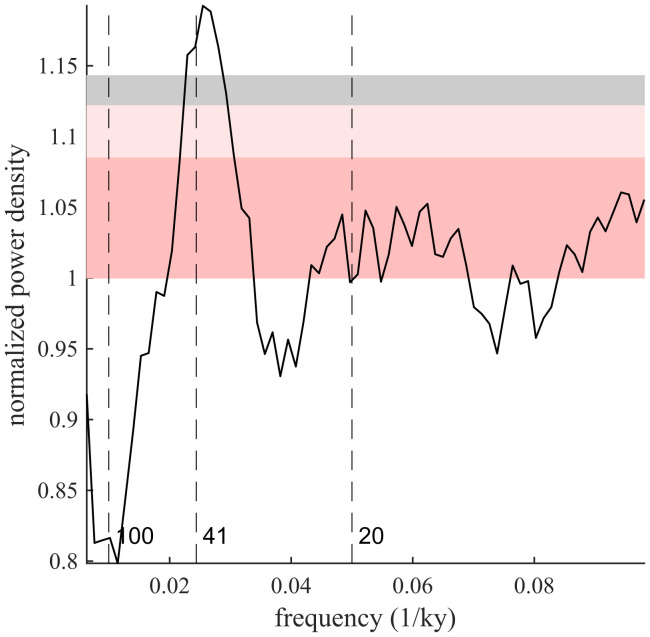
Spectral estimate for fast-spreading profiles. Individual multitaper spectral estimates from 39 ridge flank bathymetry profiles having an estimated spreading rate greater than 4 cm/y are averaged. Profiles come from regions 1 to 4, 6, 9, 11, 12, 14, and 15, with 25 of the 39 profiles coming from the East Pacific Rise (*SI Appendix*, Table S1). Each estimate is made using a multitaper procedure with a time-bandwidth product of 5, after interpolating to 5-ky resolution and prewhitening using an autoregressive order-2 and moving-average order-2 fit. This formulation produces the lowest residual variance relative to the expected variance of all examined approaches that do not involve time differencing. Similar results are obtained under a wide variety of other specifications (*Materials and Methods*). CIs are computed according to a gamma distribution for significance levels of 0.05 (pink), 0.01 (light pink), and 0.01 after a Bonferroni correction that accounts for the examination of bands associated with 100 ky, obliquity, and climatic precession found in late-Pleistocene variations in sea level (gray).

An average spectral estimate formed from spectral analysis of all ridge flank bathymetry profiles whose spreading rate exceeds 4 cm/y, 39 in total (*SI Appendix*, Table S1), is shown in Fig. 3. The excess spectral energy at the 1/(41-ky) obliquity frequency is highly statistically significant (*P* < 0.01). To examine whether this result is influenced by plausible choices with respect to prewhitening and spectral estimation, we evaluated 72 different formulations of the analysis involving the degree of prewhitening, resolution of time-series interpolation, and number of tapers applied in the multitaper method (*SI Appendix*, Table S2). Of the 72, 66 indicate that the 1/(41-ky) spectral energy is highly statistically significant (*P* < 0.01) and 71 indicate significance (*P* < 0.05), showing that the results in Fig. 3 are robust. Furthermore, if only the formulations of the spectral analysis that produce a residual spectral variance (31) that is within 20% of expectations are included, all show a statistically significant obliquity peak (*P* < 0.05).

## 3. Discussion

The FLAC simulations correspond well with observations from ocean ridges in the framework of the sea-level hypothesis (Fig. 2). At slow-spreading centers, abyssal-hill spacing varies inversely with spreading rate in response to thickening lithosphere, consistent with expectations (25).

Results at intermediate- and faster-spreading rates are diagnostic of the sea-level hypothesis and contrast with the proposal for constant spacing between abyssal hills at half-spreading rates greater than ∼3 cm/y (24). The prediction of constant spacing is based on a simplified scaling relationship wherein faults are assumed to initiate at the ridge axis, move away at a rate slower than the half-spreading rate because of accommodation by fault heave, and then become inactive at a fixed distance away from the ridge (2, 3). Instead, spacing aligns with the 100-ky period of late-Pleistocene sea-level variations at intermediate-spreading rates, and at fast-spreading rates spacing aligns with a 41-ky period. The transition from 100- to 41-ky spacing occurs at 3.8 cm/y (Fig. 2), consistent with the threshold above which faults consistently initiate in response to 41-ky magma cycles in FLAC.

Onset of 41-ky scaling may also reflect the admittance of the ridge-magmatic system to different frequencies of sea-level forcing. Admittance involves a trade-off between more rapid changes in sea level causing larger fluctuations in melt generation and an anomaly needing to be of longer duration to rise through the melting regime and be expressed at the ridge axis (12, 28). Melt reservoirs at fast-spreading ridges could further damp fluctuations of melt supply (24). Profiles from fast-spreading ridges exhibit 41-ky period variability as opposed to 100-ky variability, consistent with faster spreading allowing shorter-period anomalies in melt to ascend more quickly through the mantle.

The existence of a spreading-rate threshold that varies with parameters such as the period of magmatic forcing is relevant in evaluating earlier reports that FLAC does not produce 41-ky scaling for a 3.3-cm/y half-spreading rate (24). Although such scaling initiates only at 3.8 cm/y in our main simulations (Fig. 2), 41-ky spacing can initiate at a spreading rate as slow as 3 cm/y using the FLAC model with other, plausible parameter values (*SI Appendix*, Fig. S6). The 100-ky fault pacing is simulated in FLAC for half-spreading rates of >3 cm/y over the full range of parameter values that we consider. The exact spreading-rate threshold above which sea-level variations may influence bathymetry thus has some uncertainty.

A significant spectral peak centered on the 1/(41-ky) frequency in fast-spreading regions reinforces the finding of obliquity control (Fig. 3), but climatic-precession variations at 19- to 23-ky periods are not identified in our average spectral results. Climatic-precession fluctuations may be too rapid to consistently influence melt delivery or propagate through a melt reservoir (24). Although the technique we use is insensitive to age offsets because results are averaged in the spectral domain rather than the time domain, spectral features may be obscured through distortions of the inferred age–distance relationship. Spectral peaks at higher frequencies are more readily obscured by such distortions (32).

There are a number of potential complexities in the age–distance relationships among faults. First, uncertainties in identifying the location of zero-age crust and the location of the Brunhes–Matuyama magnetic reversal will stretch or squeeze an age model, leading to shifts in the frequency at which a spectral peak appears. Such frequency shifting may contribute to the width of the 41-ky peak in Fig. 3. Second, small ridge jumps and offset traces on ridge flanks (33) will cause offsets in an age–distance relationship from one ridge flank bathymetry profile to the next. Changes in spreading direction (34) could also distort an age scale that is defined as linearly increasing with distance from the ridge axis. Third, faulting intermittently accommodating portions of crustal extension could influence the relationship between age and off-axis distance (2). Finally, there may be a nonlinear relationship between faulting and glacial cycles because of intermittency in faulting and irregularities in sea-level variations. At slow-spreading regions multiple glacial cycles may elapse before faults are triggered and, even at faster-spreading regions, irregular variations in sea level may lead to only a subset of deglacial events being associated with faulting. A more complete analysis would track the age of individual faults and account for the possibility that faults are nonlinearly paced by variations in sea level, but this is beyond our present scope.

Several studies came to conclusions that differ from ours for reasons that we suggest relate to methods and data selection. One study found no evidence for covariation among widely spaced ridge flank bathymetry profiles (35). We suggest that covariance is difficult to detect regardless of whether abyssal hills reflect sea-level period variability because of the age uncertainties described above. Second, the 41-ky response that we observe for fast-spreading regions differs from another study (36) that reported 100-ky variations in bathymetry from the Southern East Pacific Rise. In the earlier analysis a filter was applied that removed variability at periods longer than 150 ky, which accentuates 100-ky variability given the red background spectrum that is characteristic of bathymetry variations. Because of the diverse sources of age uncertainty, there is further need to develop methods for identification of periodic signals potentially embedded in the strong background variability associated with midocean ridge bathymetry.

Our results also have implications for bathymetry during earlier geologic epochs as a consequence of differences in glacial and sea-level variability. Glacial variability during the Pliocene and early Pleistocene contains little 100-ky variability relative to the late-Pleistocene epoch that we focus on (37). If 100-ky variability in surface bathymetry or crustal thickness (21) is driven by 100-ky variations in sea level during the late Pleistocene, its amplitude should be smaller during earlier geologic epochs. A transition in bathymetric variability across the Pleistocene appears to be present in select ridge flank bathymetry profiles from the Chile Ridge (22). At still earlier times, the warmer climate of the Eocene and Miocene appears not to have experienced major changes in glaciation and sea level at orbital or 100-ky periods (38). It is then consistent with the sea-level hypothesis that an analysis of bathymetry on the flank of the Southeast Indian Ridge between 11 and 23 Ma found no evidence for quasiperiodic variations matching those of the late Pleistocene (10).

In addition to glacial cycles, many ridge processes must influence the bathymetric fabric of the sea floor. For instance, we find substantial variations in the characteristic spacing of abyssal hills in regions along the Southeast Indian Ridge (Fig. 3), and this spacing has been shown to covary with the presence of an axial valley or axial high (25) that reflects differences in magma supply rates (39). As another example, bathymetry analyzed along the Chile Rise shows Milankovitch-period variability near the edges of the ridge segment but not in the interior, possibly because more abundant magma supply at the center of the ridge segment overwhelms other sources of bathymetric variability (22). There are many potential nuances in relationships among sea level, melt supply, and bathymetry associated with diverse causes of variation in melt supply, including storage in magma chambers and off-axis volcanism, as well as variable ridge offsets (40). Our findings that glacially modulated sea-level change contributes to the bathymetric fabric of the sea floor may allow further insights into the processes that contribute to near-ridge bathymetry. In particular, interactions among magma supply, dike intrusion, and faulting (41, 42) warrant further examination.

## 4. Materials and Methods

### A. FLAC Simulations.

We run FLAC using the same parameter values as those in an earlier study (24) to reduce ambiguity in comparing results (Table 2). The domain size of our FLAC simulation is 240 km wide and 15 km deep, with spatial resolution increasing toward the ridge axis at the center of the domain (*SI Appendix*, Fig. S2). Magmatic extension where melt supply entirely fills the space created by spreading occurs for 85% of a cycle. The remaining 15% of a cycle is an amagmatic phase where plate separation must be accommodated by extension and faulting. Plate extension results in accumulation of stress and, ultimately, plastic failure along a fault according to a Mohr–Coulomb failure criterion (3). The 85 to 15% split between magmatic and amagmatic phases corresponds with the sawtooth nature of late-Pleistocene glacial cycles having a long period of lowering sea level punctuated by a rapid rise (*SI Appendix*, Fig. S1). Sawtooth variations in sea level are expected to give relatively brief intervals of reduced rates of mantle depressurization and melt supply.

FLAC is run 40 times for both 100- and 40-ky magmatic cycles. The 100-ky cycle has magmatic spreading for 85 ky and extension and faulting during the other 15 ky; the 41-ky period runs have magmatism and extension intervals of 35 and 6 ky. Abyssal-hill spacing in FLAC is quantified as the average distance between bathymetric peaks in the surface layer of the lithosphere (*SI Appendix*, Fig. S1). Profiles are split for separate evaluation of peaks on each side of the axis and trimmed to 780 ky on each side for consistency with the interval over which observations are analyzed.

FLAC fault spacing shown in Fig. 2 is computed as the mean difference between local bathymetric maxima. Local maxima are identified according to a prominence statistic defined as the difference in height between a peak and a minimum. The minimum is chosen as the higher of the nearest minima on either side of a peak. If prominence is 25 m or greater, the peak is accepted. The first identified fault on each side of the ridge is removed in simulations to ensure that an axial high is not mistaken as a fault. Note that, although useful for analyzing model simulations, the prominence method is poorly suited for identifying abyssal hills in the observed bathymetry because of its sensitivity to noise.

To examine the sensitivity of the FLAC results to different, plausible parameterizations we varied five important parameters according to suggestions in the literature. First, injection-zone widths are varied from 800 to 2,800 m, consistent with values used elsewhere (24, 43). Second, the fraction of a cycle spent in a magmatic phase, *M*, is varied between 0.65 and 0.9, consistent with the estimate of *M* values specified at intermediate- and fast-spreading ridges in an earlier study (24). Third, thermal diffusivity associated with hydrothermal circulation is varied according to the Nusselt number, *n*, or the ratio of convective to conductive heat transfer, over select values ranging between 6 and 20 for the lower crust and between 20 and 30 for the upper crust (43, 44). Fourth, the maximum viscosity in the injection zone is uncertain (4, 24, 39, 43) and is varied over values ranging from 10^18^ to 10^26^ Pa ⋅s. Finally, we consider the healing parameter $\tau_{\text{heal}}$, which controls the timescale over which the loss of cohesion at a fault caused by strain weakening recovers. We explore timescales of $0.3\tau_{f}$ to $3\tau_{f}$, where *τ_f_* is either 41 or 100 ky, because fault initiation will depend on how much stress accumulates during a forcing cycle.

For each set of parameters, 40 FLAC runs are conducted over a range of spreading rates (*SI Appendix*, Fig. S6). The same basic structure is found across the range of parameter values with abyssal-hill spacing decreasing with spreading rate until becoming locked into the period of the magmatic cycle. The conclusion that FLAC produces abyssal-hill spacing that can be controlled by magma supply variations modulated by sea level is thus a robust result.

### B. Bathymetry Selection and Age Models.

Bathymetry and magnetic data are obtained from the Marine Geoscience Data System (https://www.marine-geo.org/) in all but two cases. First, the magnetic data from ref. 45 are used to specify the location of magnetic reversals in region 17. Second, we use magnetic data from the Southern East Pacific Rise (region 4) that was reprocessed to deskew anomalies associated with tilted, magnetized blocks (46).

All ridge transects that have collocated magnetic data with a clearly identifiable Brunhes–Matuyama (B-M) magnetic reversal are analyzed. Each transect is divided into two ridge flank bathymetry profiles. Profiles are excluded only if they feature transform faults, areas of off-axis seamounts and their volcanic aprons, or regions of rotated and disrupted crust associated with migrating ridge discontinuities. See *SI Appendix*, Figs. S7–S23 and Table S1 for more detailed regional information. To better ensure high quality and resolution within our dataset, we use center-beam data extracted from multibeam swath files rather than gridded data. The average spatial resolution of individual ridge flank bathymetry profiles ranges between 25 and 90 m.

Time is linearly interpolated with distance between the ridge axis, which is visually identified, and the B-M magnetic reversal, which is assigned an age of 780 ky (29). Magnetic intensity series are first filtered between 1/(200 km) and 1/(5 km) to isolate the reversal from long-wavelength trends and short-term anomalies not associated with a reversal. Pairs of maximum and minimum extreme values of filtered magnetic series are identified and differenced. Differences that exceed 30% of the fraction of the range are selected as candidate magnetic reversals, with the pair flanking nearest to the ridge axis taken as B-M magnetic reversals. Two regions have more variable magnetics relative to their range and are assigned different thresholds: 50% for EW9708 and 40% for WEST09MVe. The precise location of the B-M magnetic reversal is specified as the midpoint between a paired maximum and minimum.

Estimated distances of the B-M reversals to the ridge axis are compared in regions with three or more ridge flank bathymetry profiles on a single side of the ridge axis, and outliers exceeding 3 SDs from the median distance are excluded. Although our spreading estimates are generally consistent with those from plate-motion models (47), use of coacquired magnetics for age control minimizes the effects of geologic complexity such as unidentified ridge jumps as well as local navigational uncertainties.

Distance between the position of each bathymetry measurement and the ridge axis is measured perpendicular to the local ridge axis. Rhumb lines are used for simplicity and because the distinction between these and the small circles defining plate motion is negligible over the several-kilometer distances between the ridge axis and B-M reversals. The strike of the ridge axis is defined as the median value of the directions between all successive ridge axis picks, with the local ridge axis shifted to pass through each axis pick. The ridge axis is thus approximated as trending along a constant strike with offsets insomuch as ridge picks are not aligned.

### C. Characteristic Wavelength.

The distribution of variability in abyssal-hill bathymetry has been described as stochastic—for example, as following a Matérn process (25)—or also as including quasiperiodic components (12). We seek to estimate a characteristic wavelength that is applicable under either scenario. Our technique is to estimate the spectral density of the derivative of each bathymetric profile in a region, average individual regional spectral estimates together, and find the frequency with maximum spectral density. Spectral analysis is applied to each bathymetry profile using the multitaper method with seven tapers (48). Before computing spectral power, time series are linearly interpolated to even spacing. Averaging together the spectral estimates from each profile gives a more stable and general description.

The utility of taking the derivative of the bathymetry can be illustrated for both the purely stochastic and quasiperiodic scenarios. The power spectrum of a Matérn process is

[1] $$P(\lambda )=\frac{\sigma }{\lambda_{m}\sqrt{\pi }}\frac{1}{{\left(1+{\left(\lambda /\lambda_{m}\right)}^{2}\right)}^{\nu }},$$

where *σ* defines the amplitude. *λ* is wavelength, and *λ_m_* is the corner wavelength that controls where *P* transitions from increasing toward longer wavelengths following a power law of *ν* to having constant energy with wavelength.

The spectrum of the derivative of bathymetry that follows a Matérn process is Eq. 1 multiplied by $\lambda^{2}$. The maximum of the resulting equation, found by setting the derivative equal to zero, is $\lambda_{\text{max}}=\lambda_{m}{\left(\nu -1\right)}^{\left(-1/2\right)}$. As long as *ν* is near 2, as has typically been found for midocean ridge bathymetry (26), the maximum is a good indicator of *λ_m_*.

Alternatively, bathymetry may combine a quasiperiodic and stochastic process. Consider

[2] $$P(\lambda )=a\delta_{\left(\lambda ,\lambda_{o}\right)}+\sigma \lambda^{-\nu },$$

where *a* is the spectral amplitude at a particular wavelength, *λ_o_*, and $\delta_{\left(\lambda ,\lambda_{o}\right)}$ equals zero otherwise. If *ν* is again ∼2, taking the derivative of the bathymetry levels the background spectrum and facilitates identifying the spectral peak associated with *λ_o_* as the maximum (30). This approach appears applicable in practice because average regional spectra of bathymetry gradients show a structure with well-defined maxima (*SI Appendix*, Fig. S4).

### D. Testing for Spectral Peaks.

As for the characteristic wavelength, frequency spectra are also estimated using the multitaper method (48) but after converting off-axis distance to time. We are concerned specifically with evaluating the statistical significance of spectral peaks at frequency bands containing 1/(100 ky) or 1/(41 ky), in keeping with the scaling of characteristic wavelengths with spreading rate (Fig. 2). A common null model is an autoregressive (AR) order-one process (1) (49). More-detailed null models include integrated and moving averages (50), portions of the sample autocorrelation function (30), and power-law distributions (51).

Each of these null models implies an operation for whitening the spectral estimate or making the distribution of spectral energy approximately level across frequencies, either by dividing the spectral estimate by the null model in the frequency domain or by performing an equivalent operation in the time domain. Whitening or leveling spectral estimates also improves the power of statistical tests for identifying spectral peaks (30) and was used in foregoing statistical analyses of near-ridge bathymetry (12, 34, 52).

A level spectrum is expected to follow a gamma distribution whose shape parameter, *k*, is equal to half the degrees of freedom in the spectral estimate. The scale parameter, *θ*, equals $\sigma^{2}/k$, where $\sigma^{2}$ equals both the variance of the bathymetry and, in the normalization convention that we use, the mean of the spectral estimate. A metric was recently proposed for purposes of evaluating the fit of a null model to a spectral estimate (31). The expected variance of the logarithm of a leveled spectrum is $\Psi_{3}(k)$, where $\Psi_{3}$ is the trigamma function. The fit of the null hypothesis is evaluated as the ratio of the observed to the expected variance of the logarithm of the leveled spectral estimate, *F*. An advantage to using the logarithm of the spectrum is that the variance of ln P is less sensitive to concentrations of spectral energy than *P*.

Prewhitening, or leveling, is accomplished by fitting an autoregressive moving-average (ARMA) model in the time domain and removing the estimated ARMA structure from the time series (50). Removal of a higher-order ARMA structure generally leads to a more-level spectral estimate and a lower *F*. We explore the implications of different order models by fitting ARMA models ranging from a simple autoregressive order-one model, ARMA(1,0), to an autoregressive order-two and moving-average order-two model, ARMA(2, 2).

In addition to selection of the ARMA model, there are three additional variants that we explore on account of being plausible and having precedent. We explore specifying time-bandwidth products of between 3 and 5 for the multitaper analysis, where higher time-bandwith products imply a larger number of degrees of freedom in the spectral estimate associated with a frequency band but lower frequency resolution (48). We also explore applying an initial prewhitening record consisting of taking the time derivative of the bathymetry time series. This approach was used elsewhere (12, 22) but has been criticized as potentially leading to conflation of the corner frequency in a Matérn process with a spectral peak (10, 23).

Finally, the interpolated spacing of the time series is relevant with respect to the structure that the ARMA model seeks to fit. Interpolating to higher resolution than the effective sampling of bathymetry, for example, can lead to an overly red or steep spectral estimate at the highest frequencies, whereas interpolating to lower resolution risks aliasing unresolved energy to lower frequencies (30). We explore sampling bathymetry at 1- or 5-ky intervals. To suppress aliasing, 5-ky sample spacing is achieved by first linearly interpolating a bathymetry profile in the time domain to 1-ky resolution, smoothing by convolving with a 5-ky Hamming window, and decimating the time series to 5-ky resolution.

Analysis of all combinations of ARMA models, differencing, and interpolation selection lead to 72 distinct spectral treatments for each bathymetry profile (*SI Appendix*, Table S2). The average value of *F* across the 39 bathymetry profiles associated with a half-spreading rate exceeding 4 cm/y has a minimum of 0.58 from a maximal prewhitening approach involving an ARMA(2, 2) model, differencing, and interpolation to 5-ky intervals and a maximum of 9.3 for a minimal treatment involving an ARMA(1,0) model, no differencing, and interpolation to 1-ky intervals. Both maximum and minimum cases have the same time-bandwidth product of 5, reflecting that *F* has a low sensitivity to this parameter. If a threshold half-spreading rate of 3.8 cm/y is instead used for inclusion of a ridge flank bathymetry profile, 53 profiles are included, and 61 of 72 formulations indicate highly statistically significant results (*P* < 0.01) and 69 of 72 give significant results (*P* < 0.05).

The overall spectral estimate is formed by averaging the spectral estimate associated with each of the 39 ridge flank bathymetry profiles having a half-spreading rate exceeding 4 cm/y (*SI Appendix*, Table S1). Confidence intervals are determined from a gamma distribution with shape and scale parameters respectively equal to *k* and $\sigma^{2}/k$, where k=n(2b−1), *n* is the number of spectral estimates, and *b* is the time-bandwidth product. This formulation corresponds to an assumption that noise between bathymetry profiles is independent. Also possible is to weight each region equally, such that bathymetry profiles from more heavily sampled regions are down-weighted. Such a weighted average leads to a higher power spectral density in the obliquity band in each of the 72 formulations of the test, primarily reflecting that power spectral density near 1/(41 ky) is only weakly concentrated in region 4, which accounts for 18 of the 39 bathymetry profiles.

Tests are conducted at the 1 and 5% significance levels and are one sided to evaluate whether concentrations of spectral power exceed the null distribution. Selecting only results with an *F* between 0.8 and 1.2 leads to a subgroup of 17 formulations for which the 1/(41-ky) peak is always significant (*P* < 0.05). Although many formulations also give a significant spectral peak at the 1/(100-ky) frequency, these are not pursued because of potential conflation with the corner frequency of a Matérn process (*SI Appendix*, Fig. S6).

Approaches taken elsewhere to test for significant spectral peaks treat each resolved frequency band as an independent test (10, 31). Such multiple testing does not apply in the present context, at least to the same extent, because of our a priori hypothesis that bathymetry is influenced by sea level and the fact that sea level contains significant spectral peaks only at the 1/(100-ky), obliquity, and climatic precession bands (12, 53). A Bonferroni correction can be made to account for testing three frequencies, in which case spectral power density is evaluated at the $\alpha_{B}=\alpha /3$ level (54). The statistical significance of the 1/(41-ky) peak at fast-spreading ridges (Fig. 3) is consistent whether evaluated at the α=0.01 or $\alpha_{B}=0.01/3$ level. Furthermore, there is an a priori basis for expecting slower-spreading ridges to be more sensitive to lower-frequency variations in sea level (12), such that applying this Bonferroni correction is a conservative approach to estimating significance.

## Supplementary Material

Supplementary File

## Data Availability

Data and code used in this study, along with code to reproduce the figures in the main text, are posted on the Harvard Dataverse (https://doi.org/10.7910/DVN/XKAR4H) (55). Our work relies, in part, on previously published data from Marine Geoscience Data System (https://www.marine-geo.org/index.php) (56).
