# Supplementary material for: Influence of late Pleistocene sea-level variations on midocean ridge spacing in faulting simulations and a global analysis of bathymetry
Source: Proc Natl Acad Sci U S A. 2022 Jul 7;119(28):e2204761119. doi: 10.1073/pnas.2204761119 (PMC9282452; doi:10.1073/pnas.2204761119)
Supplement: Supplementary File [file pnas.2204761119.sapp.pdf]

PNAS
www.pnas.org

Supplementary Information for

Influence of Pleistocene sea-level variations on mid-ocean ridge spacing in faulting simulations and a global analysis of bathymetry

P. Huybers, P. Liautaud, C. Proistosescu, B. Boulahanis, S.M. Carbotte, R.F. Katz, C. Langmuir

Corresponding authors: P. Huybers and C. Langmuir.
E-mails: phuybers@fas.harvard.edu and clangmuir@eps.harvard.edu

This PDF file includes:

Figs. S1 to S23 (not allowed for Brief Reports)
Tables S1 to S2 (not allowed for Brief Reports)
SI References

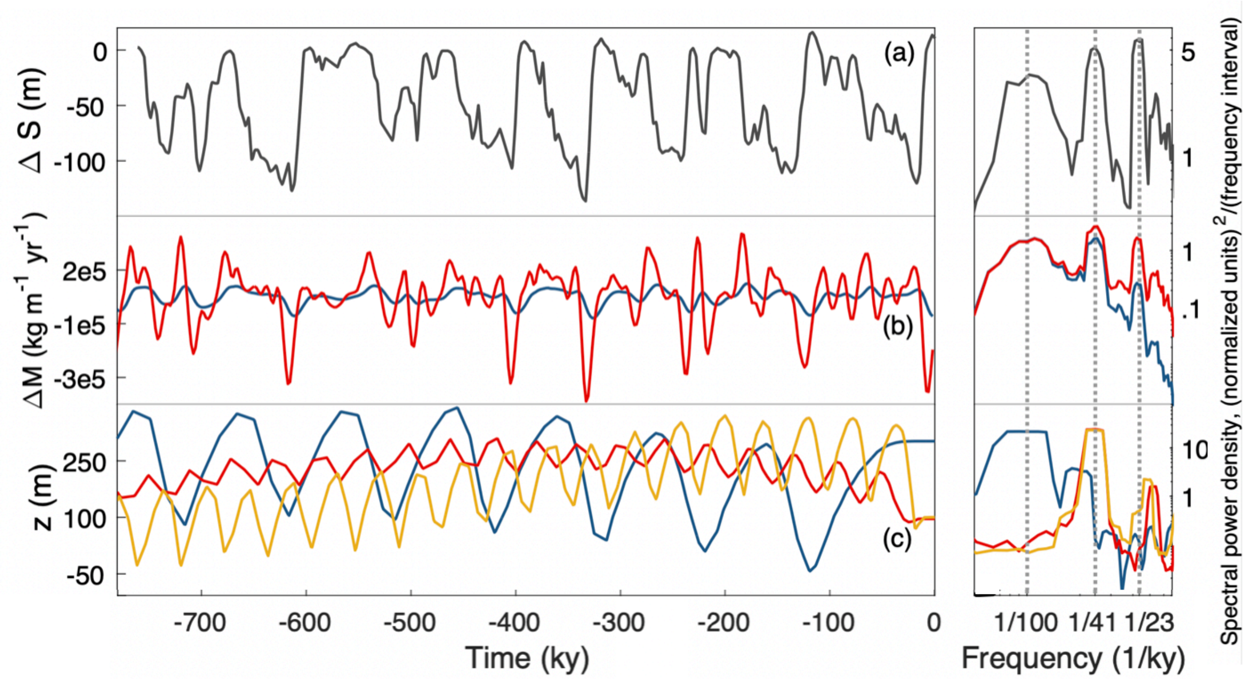

Fig. S1. Time series and spectral estimates pertaining to the sea-level driven faulting hypothesis. **(a)** Pleistocene sea-level variability (1) and a spectral estimate over 780 ka. **(b)** Estimated melt supply changes due to sea level at intermediate (2.5 cm/yr, blue) and very fast (7 cm/yr, red) spreading rates from a simple model (2), assuming a permeability of $10^{-12.5}$m^2 at 1% melt fraction. **(c)** Simulated bathymetry from a numerical faulting model (FLAC) having slower (100 kyr melt supply variations, 3.4 cm/yr, dark blue) and faster variations (41 kyr melt supply, 4.3 cm/yr in red, 6.7 cm/yr in yellow). Spectral estimates accompany each time-series, and these are normalized by mean spectral energy and, for bathymetry, include 95% confidence intervals at select frequencies (vertical bars).

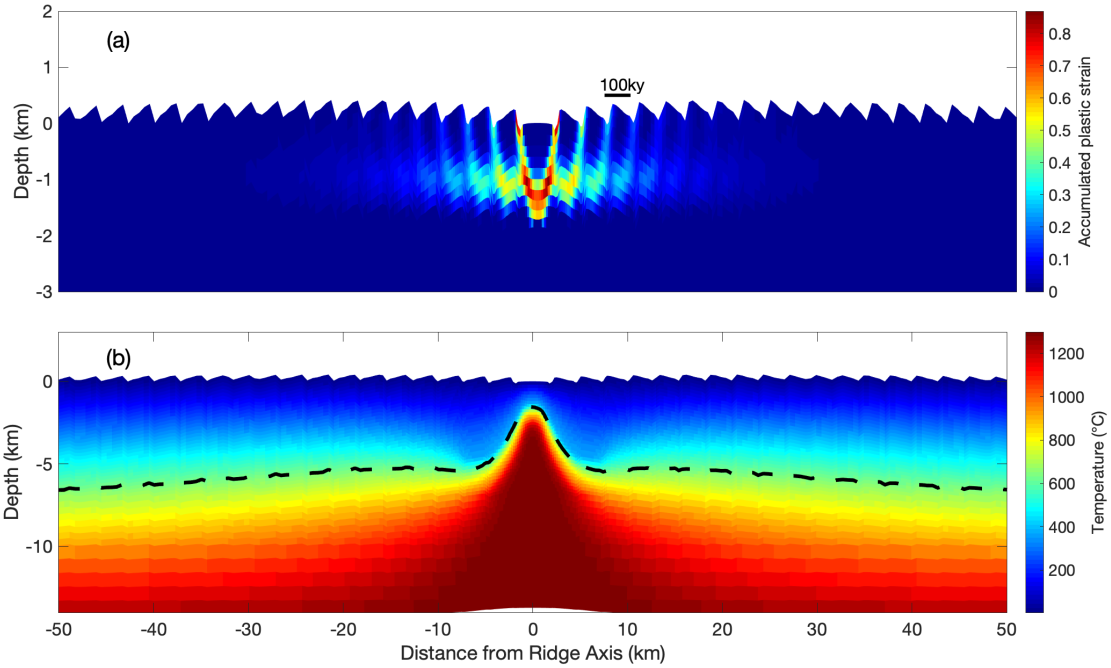

Fig. S2. Cross-sections of simulated lithosphere from FLAC with a spreading half-rate 2.7cm/year and magmatic cycling period of 100ky. (a) Accumulated plastic strain, revealing the fault zones on which extension is accommodated during amagmatic periods. (b) Simulated temperature structure of the ridge system. The black dashed line indicates the 650°C isotherm that corresponds with the lithosphere boundary.

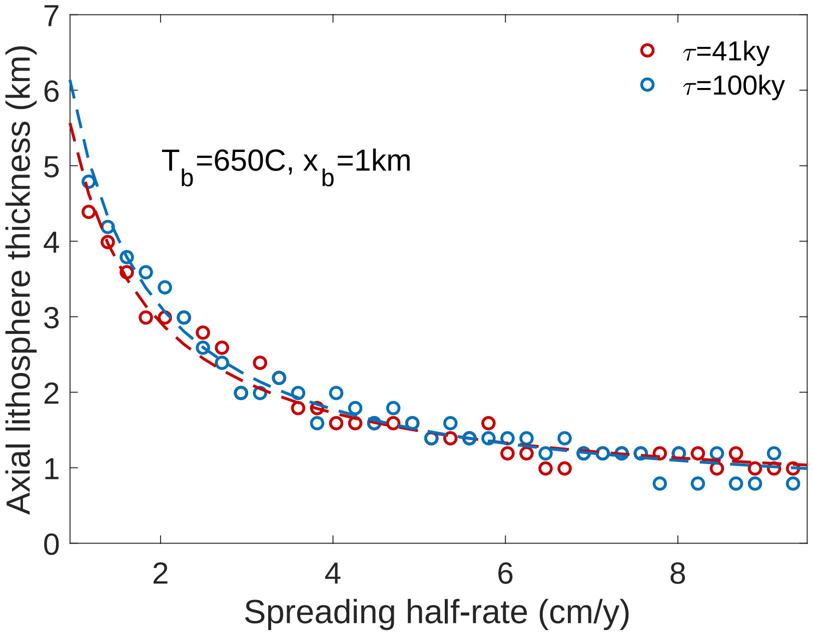

Fig. S3. Thickness of the lithosphere at the ridge axis as a function of spreading rate computed from FLAC. The base of the lithosphere is taken to be the $650°$C isotherm (T_b) and the ridge axis is taken as 100m off-axis from the edge of the injection zone (x_b). Dashed lines represent a nonlinear least-squares fit of the form $f = 0.5 + 4.8/U_0$ for the 41 kyr forcing, and $f = 0.4 + 5.4/U_0$ for the 100 kyr forcing, demonstrating that FLAC reproduces the well-known relationship (3) that the thickness of the axial lithosphere scales inversely with spreading rate.

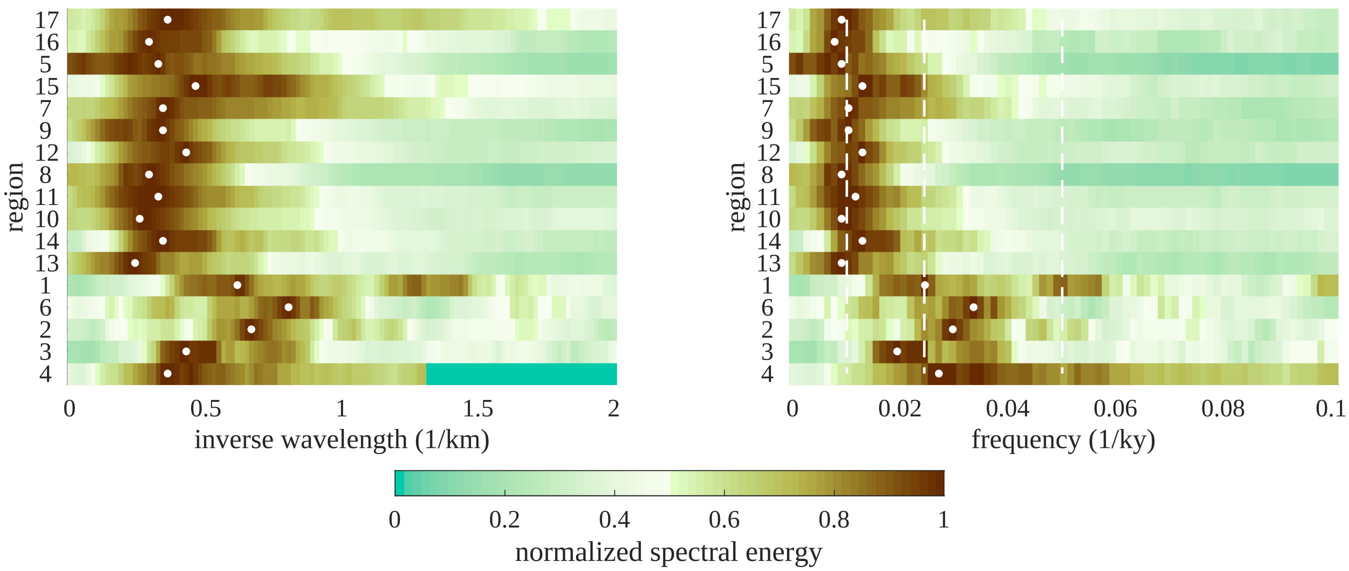

Fig. S4. Regional spectral estimates. (Left) Average spectra of the gradient of bathymetry for each region, as indicated by numbers along the y-axis. Regions are organized from slowest (top) to fastest (bottom) average half-spreading rates. Rather than the most energetic wavelength (white dots) trending toward larger inverse wavelengths, the fastest spreading regions (6, 2, 3 and 4) trend toward smaller inverse wavelengths. (Right) Computing spectra as the time-rate-of change of bathymetry shows that maxima cluster around the 1/(100 kyr) and 1/(41 kyr) frequencies (vertical white dashed lines). Regions 1 and 2 also show secondary maxima at higher frequencies consistent with the climatic precession band (1/18-1/23 kyr).

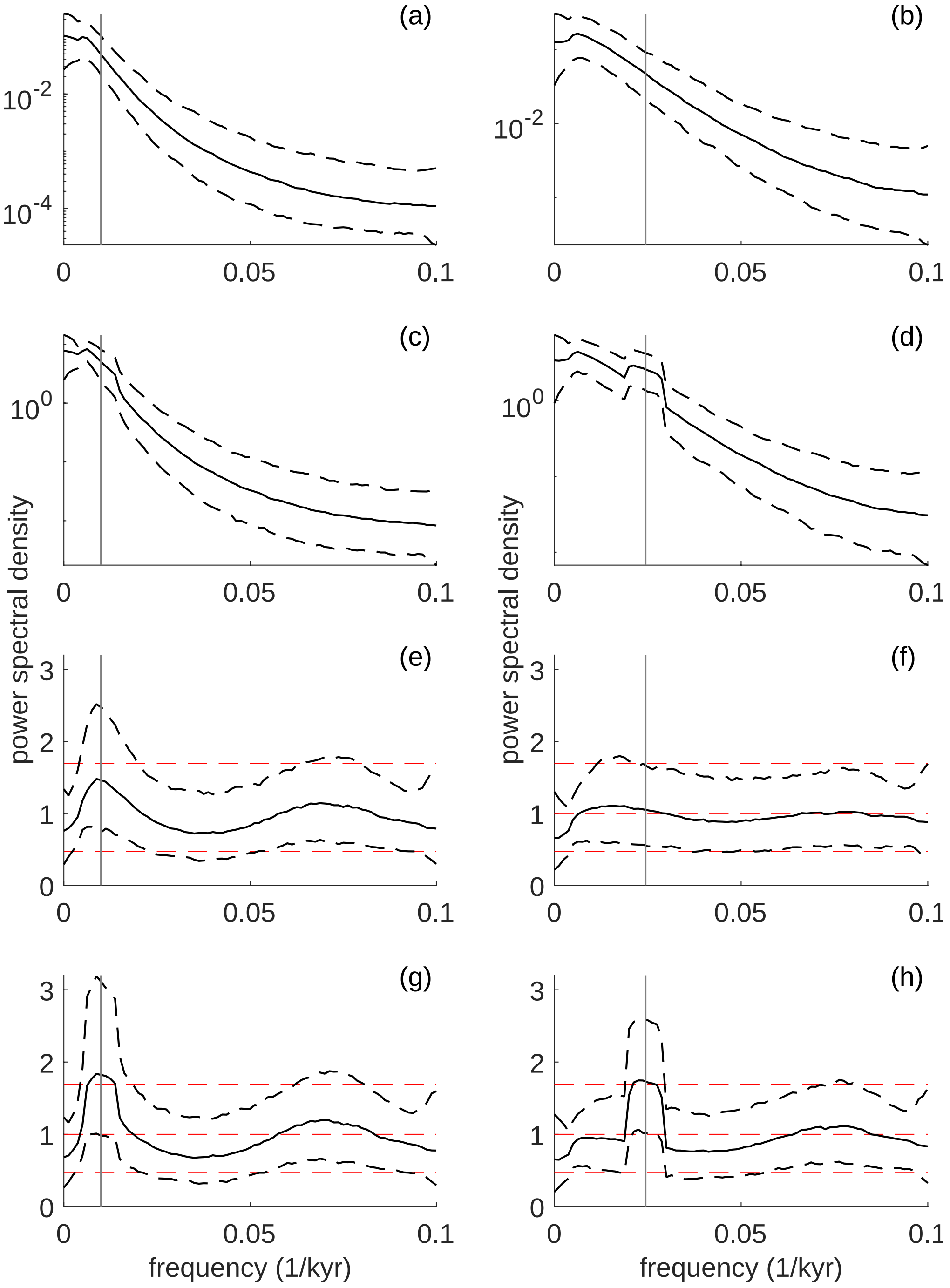

Fig. S5. Spectral estimates of Matérn processes. Matérn processes (see Eq. 1 in the main text) are realized with a power-law coefficient, ν, ranging between 1.5 and 2.5 with equal probability. Shown are the median of 1000 trials (solid black line) along with the 5th and 95th percentiles (dashed line). Panels on the left are realized from a Matérn process with a corner frequency of 1/(100 kyr) and those on the right with a corner frequency of 1/(41 kyr) (vertical gray lines). Panels **a** and **b** are from pure Matérn processes, and panels **c** and **d** are Matérn processes with, respectively, 1/(100 kyr) and 1/(41 kyr) periodic variability added with an amplitude such that the variance of the periodic component equals 25% of that of the stochastic process. The lower four panels correspond to the upper four but after applying an ARMA(1,2) prewhitening approach (4, 5). The 5th and 95th percentile theoretical confidence intervals are shown based on a gamma distribution (red dashed lines). Panel (e) shows a clear upward bias of the empirical relative to theoretical confidence intervals around 1/(100 kyr) owing to deficiencies in whitening the lowest frequencies. In contrast, panel (f) illustrates more general consistency between the whitened spectra and theoretical expectations, including around 1/(41 kyr). Note that the four upper panels are plotted using a logarithmic y-axis whereas the lower four are linear.

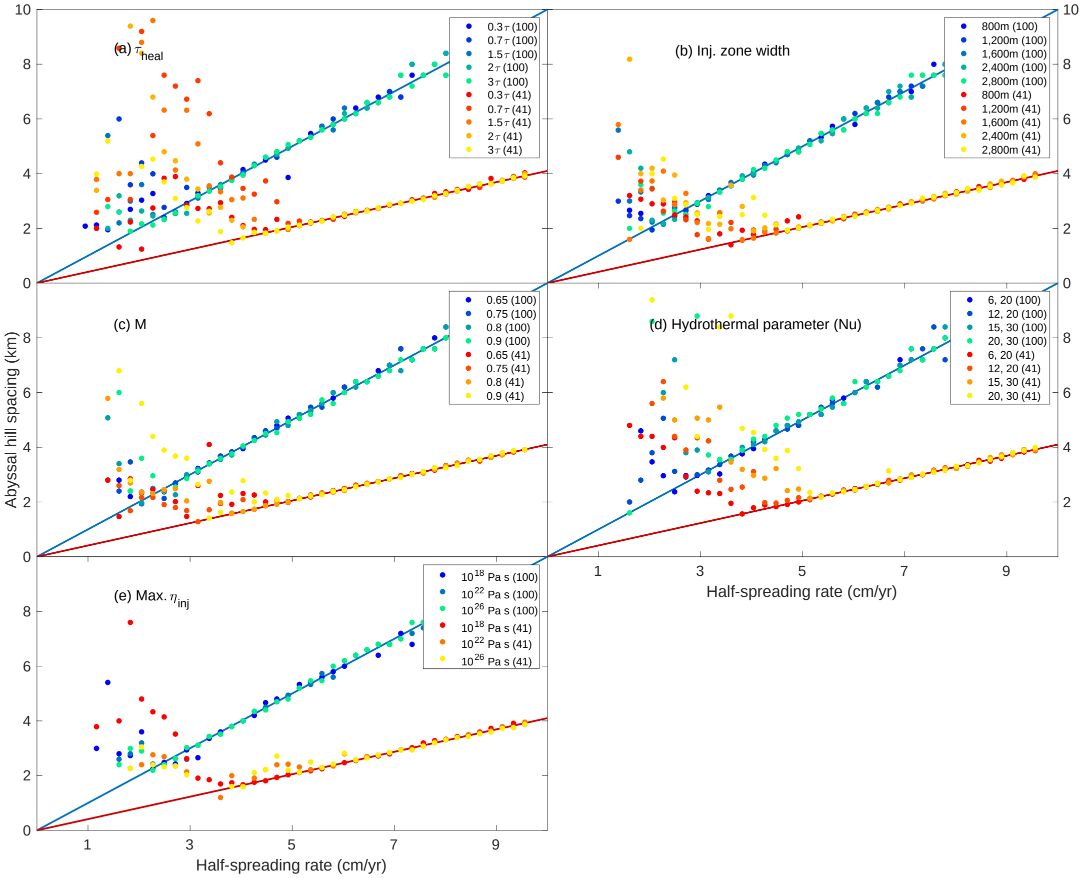

Fig. S6. Sensitivity of abyssal hill spacing to changes in FLAC parameters. Runs spanning spreading half-rates from 1 to 10 cm/yr are shown for sets of five different parameters. **(a)** Fault healing is varied between 0.3 to 3 times the magma cycle periods, where magma cycles are either 41 kyr or 100 kyr. **(b)** The width of the magmatic accretion zone is varied from 800-2800 m, where 2000 m is the default. **(c)** The fraction of plate extension accommodated by the magmatic phase is varied from 0.65-0.9, where 0.85 is the default. **(d)** The rate of cooling from near-axis hydrothermal circulation is parameterized using a Nusselt number from a lower region, ranging from 6-20, and an upper region, ranging from 20-30, where 6 and 12 are respective default values. Finally **(e)**, the maximum viscosity in the injection zone is varied from 10^{18} to 10^{26} Pa s where the default is 10^{19} Pa s. Each set of experiments is conducted once with 41 kyr period magma cycling and another time using a 100 kyr period magma cycle. Abyssal hill spacing is estimated using a peak prominence criteria. Scatter in estimated abyssal hill spacing at slower spreading rates reflects variability in fault size and resulting ambiguity in which peaks should be included when estimating spacing. These results indicate that the basic result that FLAC is paced by sea-level period fluctuations at intermediate and faster spreading rates is robust to reasonable parameter variations.

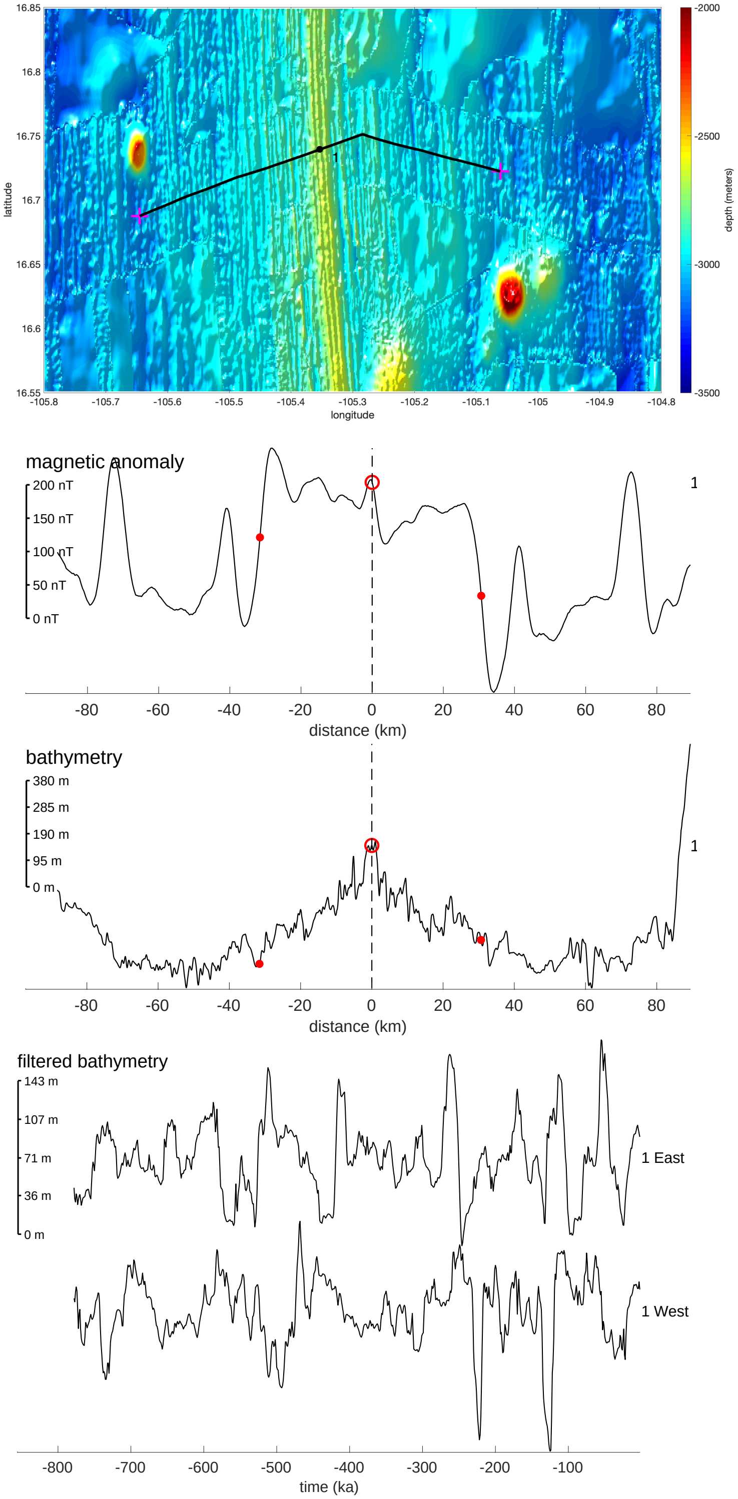

Fig. S7. Data from PANR01MV. **(a)** Bathymetric map showing a portion of the PANR01MV cruise track (black) that crosses nearly perpendicular to the ridge axis. The estimated center of the ridge is indicated by a black dot, the estimated locations of the Brunhes-Matuyama reversal by magenta crosses, and the intervening cruise track is indicated in black. **(b)** Magnetic anomaly estimates with the location of the Brunhes-Matuyama reversal (red dots) and center of the ridge axis indicated (red circle). All values are in anomaly units with a scale bar in the upper left. **(c)** Bathymetry along the cruise track. **(d)** Bathymetry plotted against time and high-pass filtered to only retain frequencies above 1/200 ky. Time is estimated linearly with off-axis distance between 0 ka at ridge axis and 780 ka at the Brunhes-Matuyama reversal.

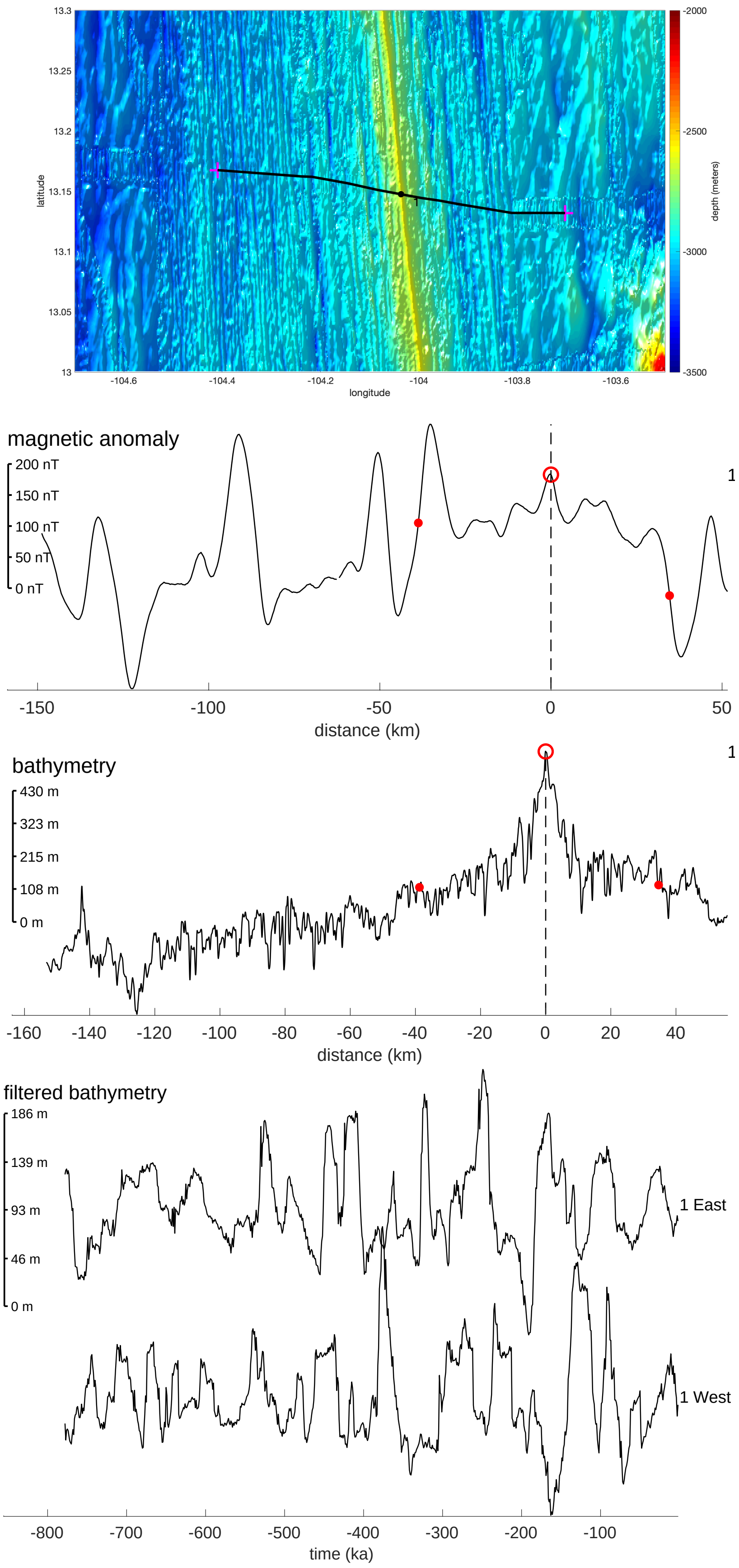

Fig. S8. Data from cruise PPTU01WT. Features as described in Fig. S7.

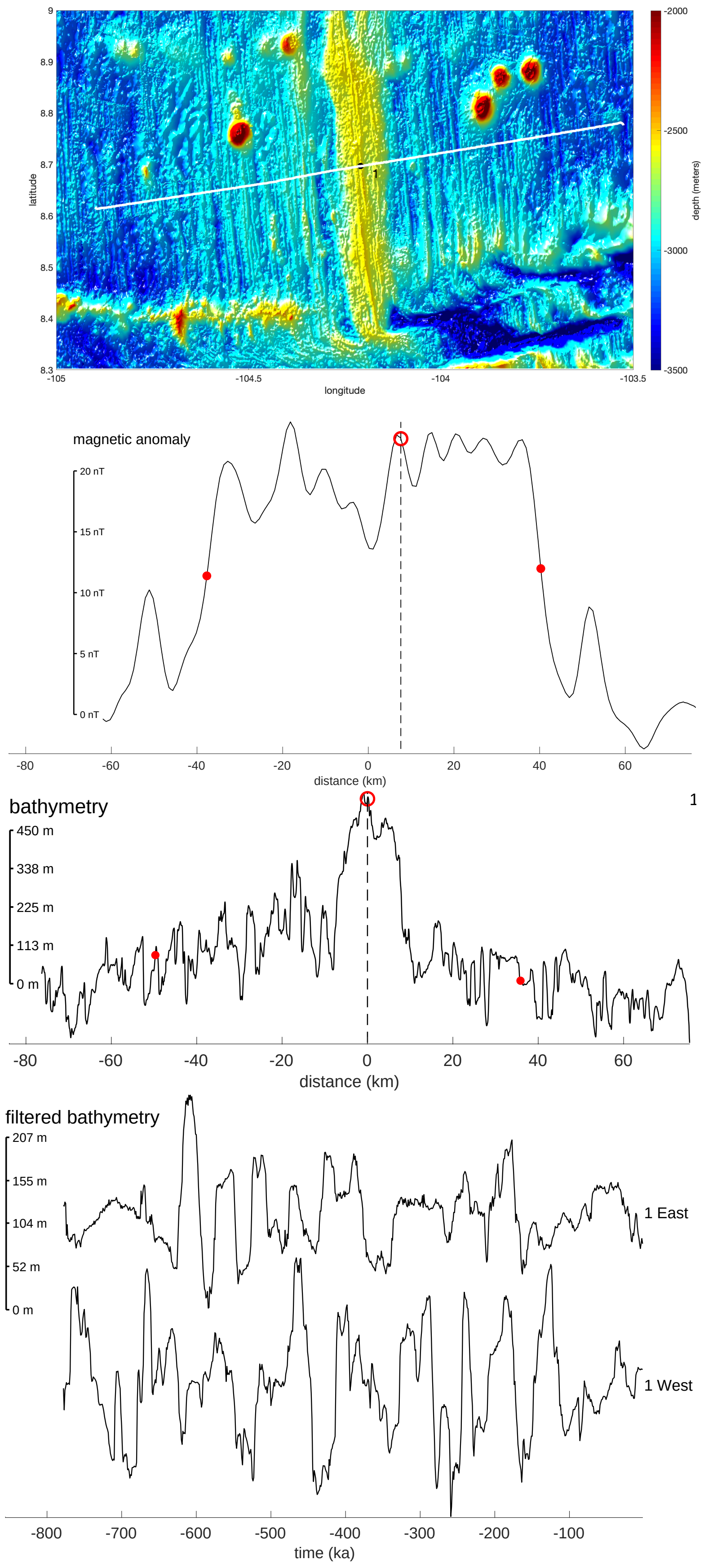

Fig. S9. Data from cruise EW9708. Features as described in Fig. S7.

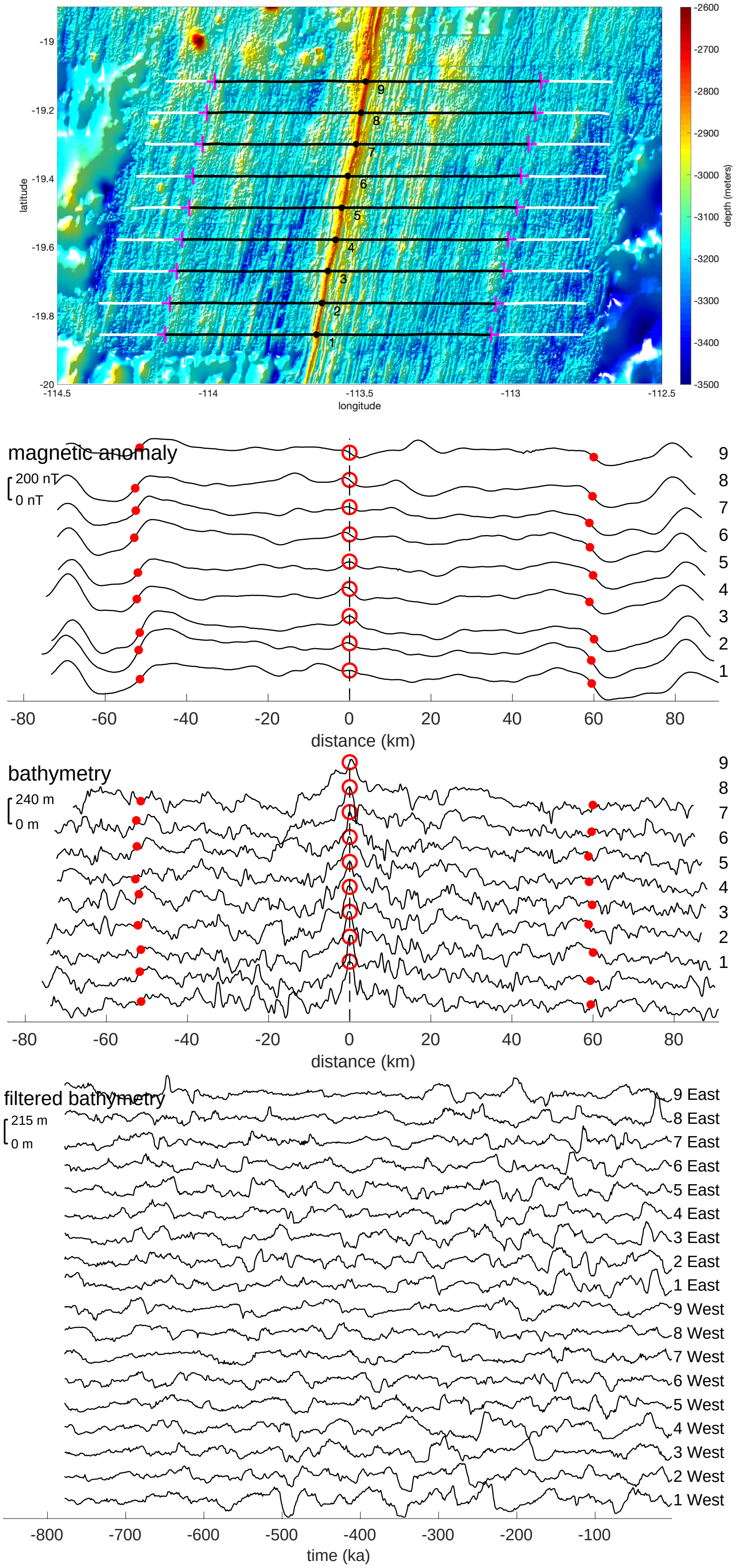

Fig. S10. Data from cruise SOJN01MV. Features as described in Fig. S7. We note that the 41 ky response that we observe at the fastest spreading rates differs from the conclusions of ref. (6), where 100 ky variations were suggested to exist on the southern East Pacific Rise. This difference may result because ref. (6) did not prewhiten the bathymetry data and applied a filter that removed variability at periods longer than 150 ky, leaving strong background variability at 100 ky.

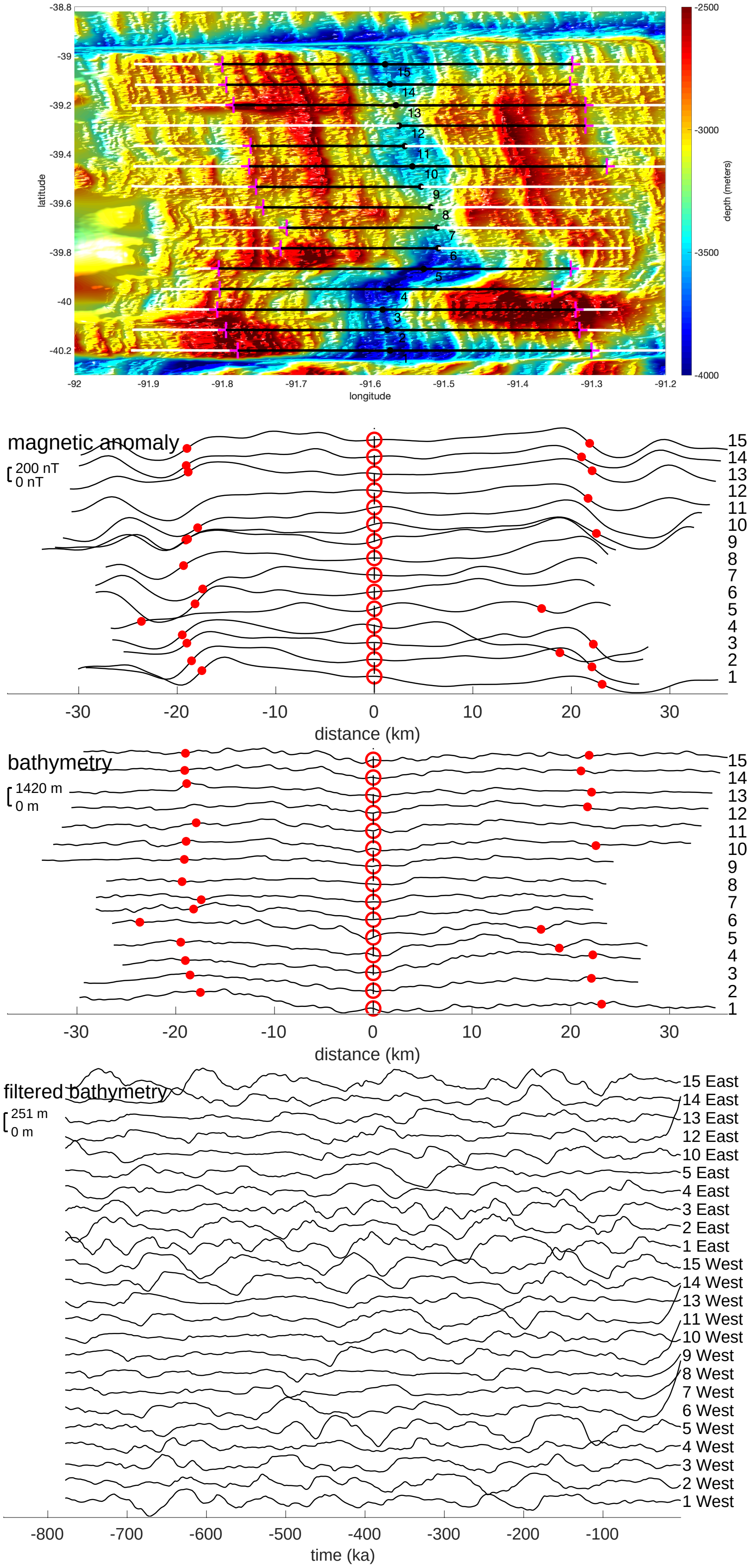

Fig. S11. Data from cruise PANR04MV. Features as described in Fig. S7.

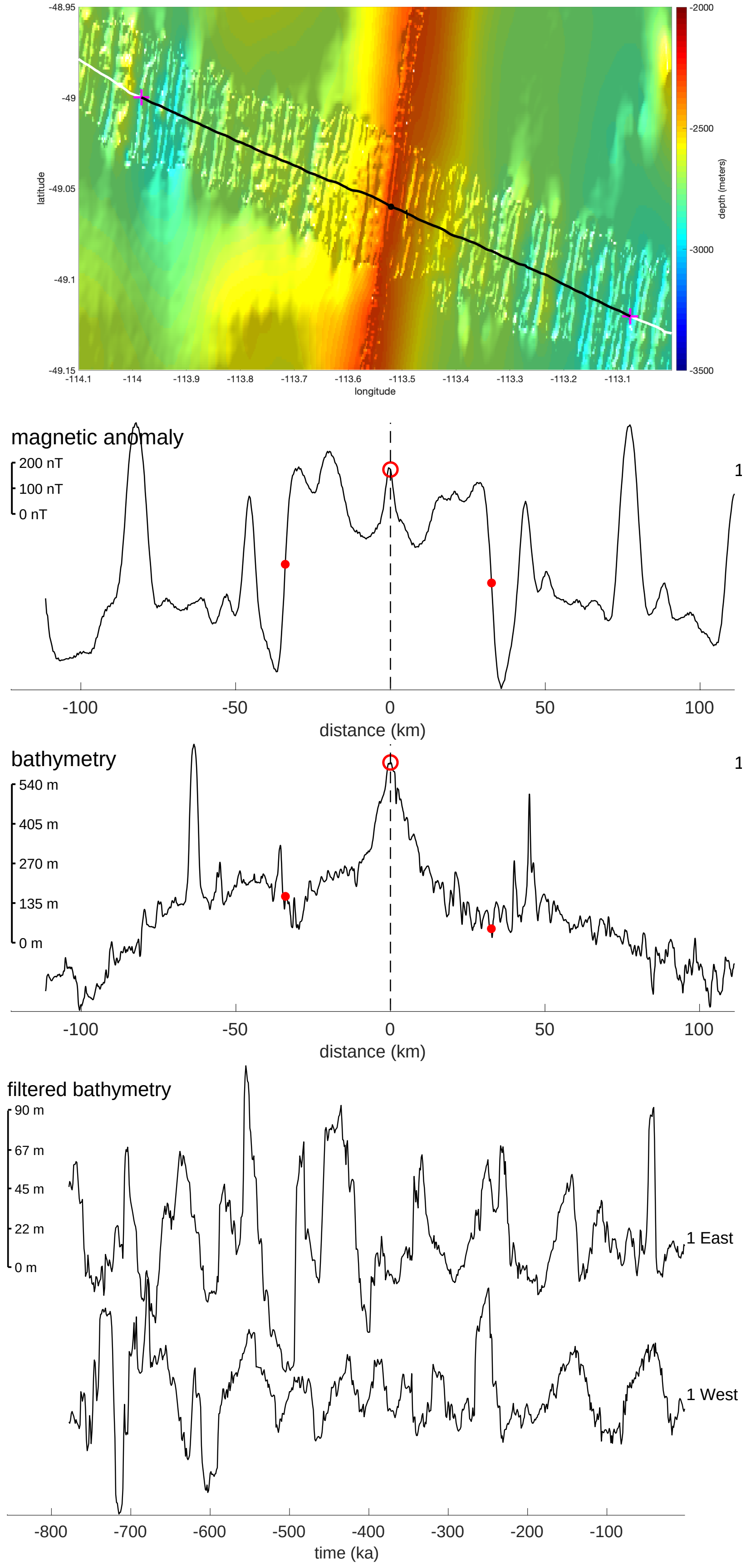

Fig. S12. Data from cruise NBP9707. Features as described in Fig. S7.

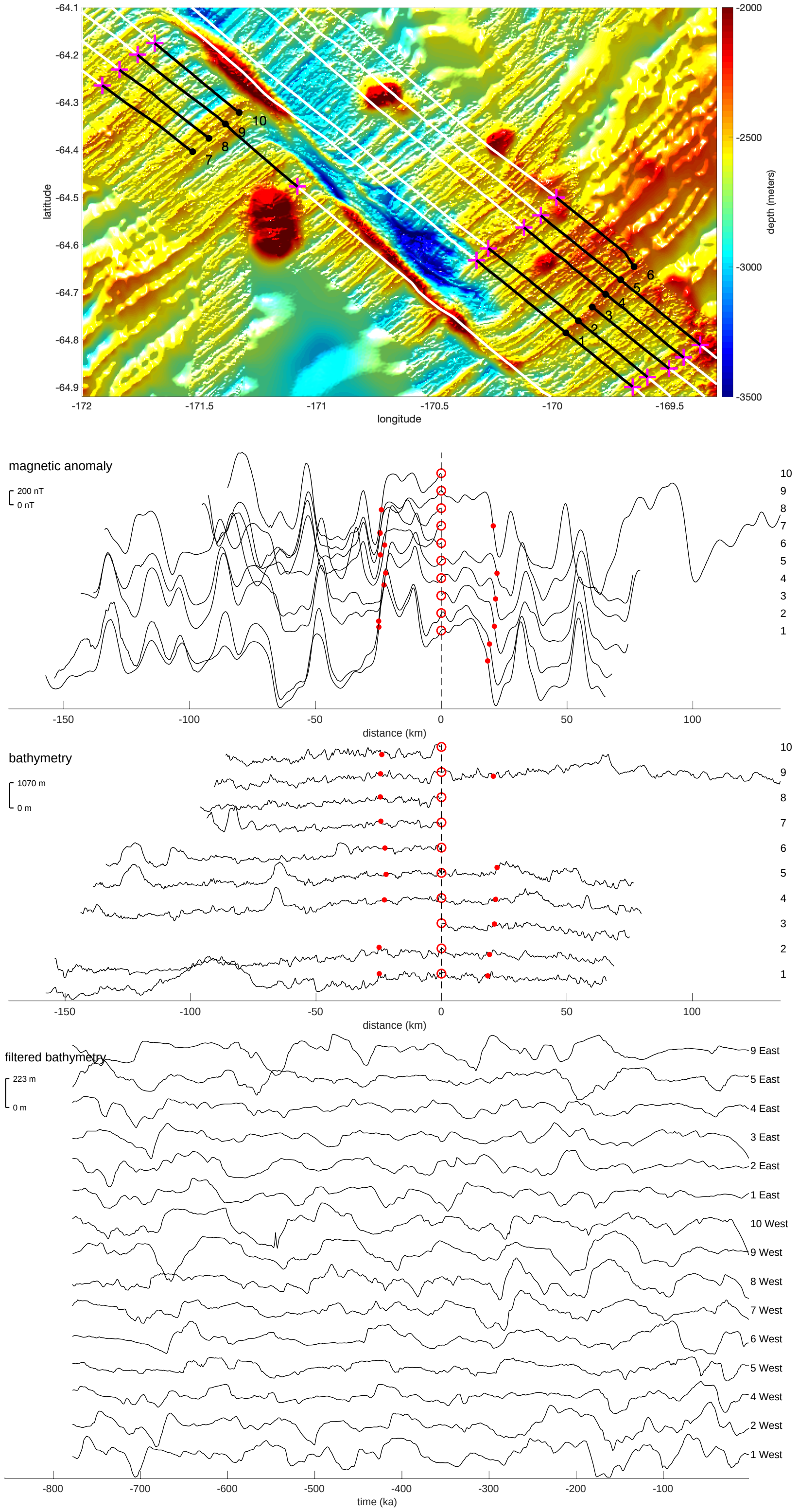

Fig. S13. Data from EW9201. Features as described in Fig. S7.

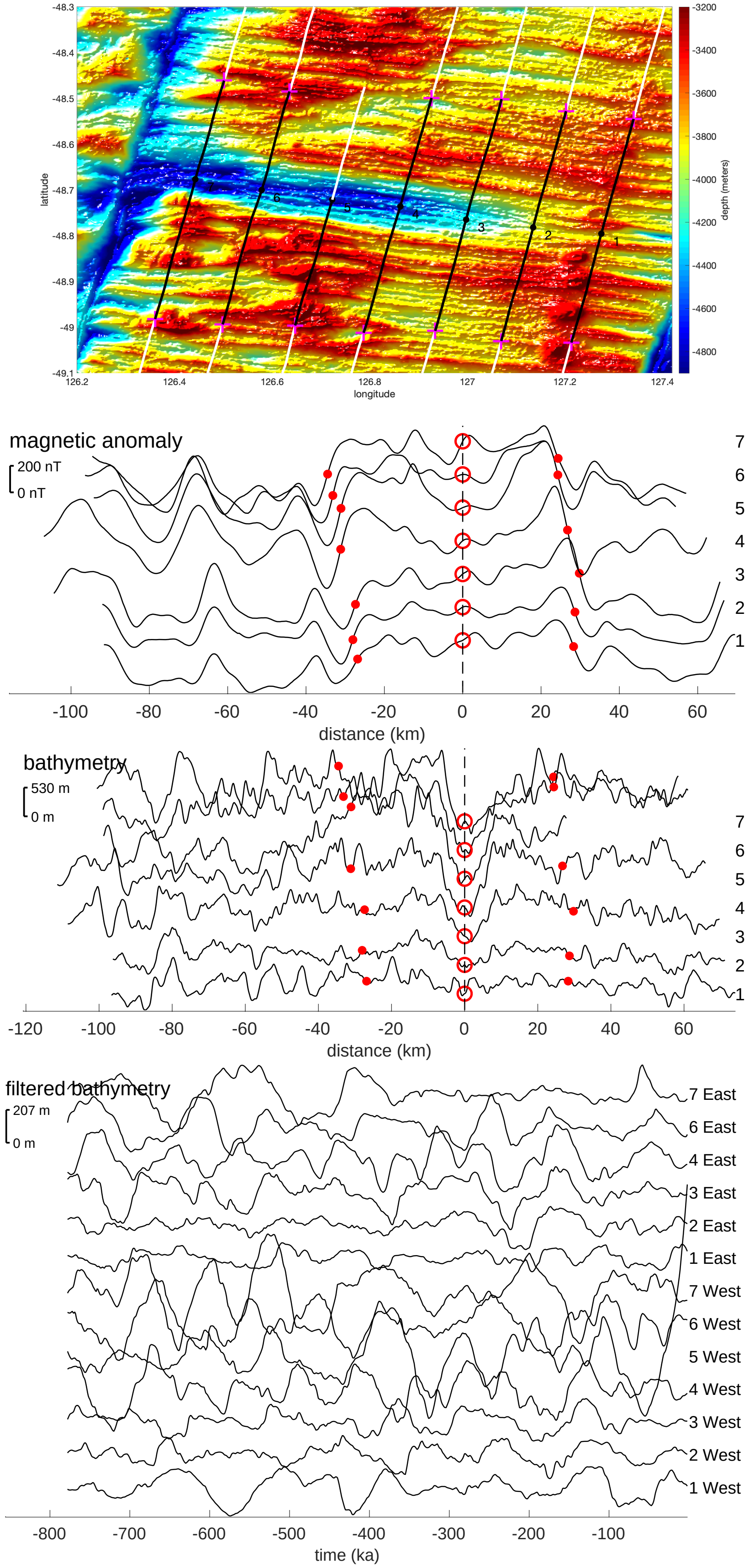

Fig. S14. Data from cruise BMRG05MV. Features as described in Fig. S7.

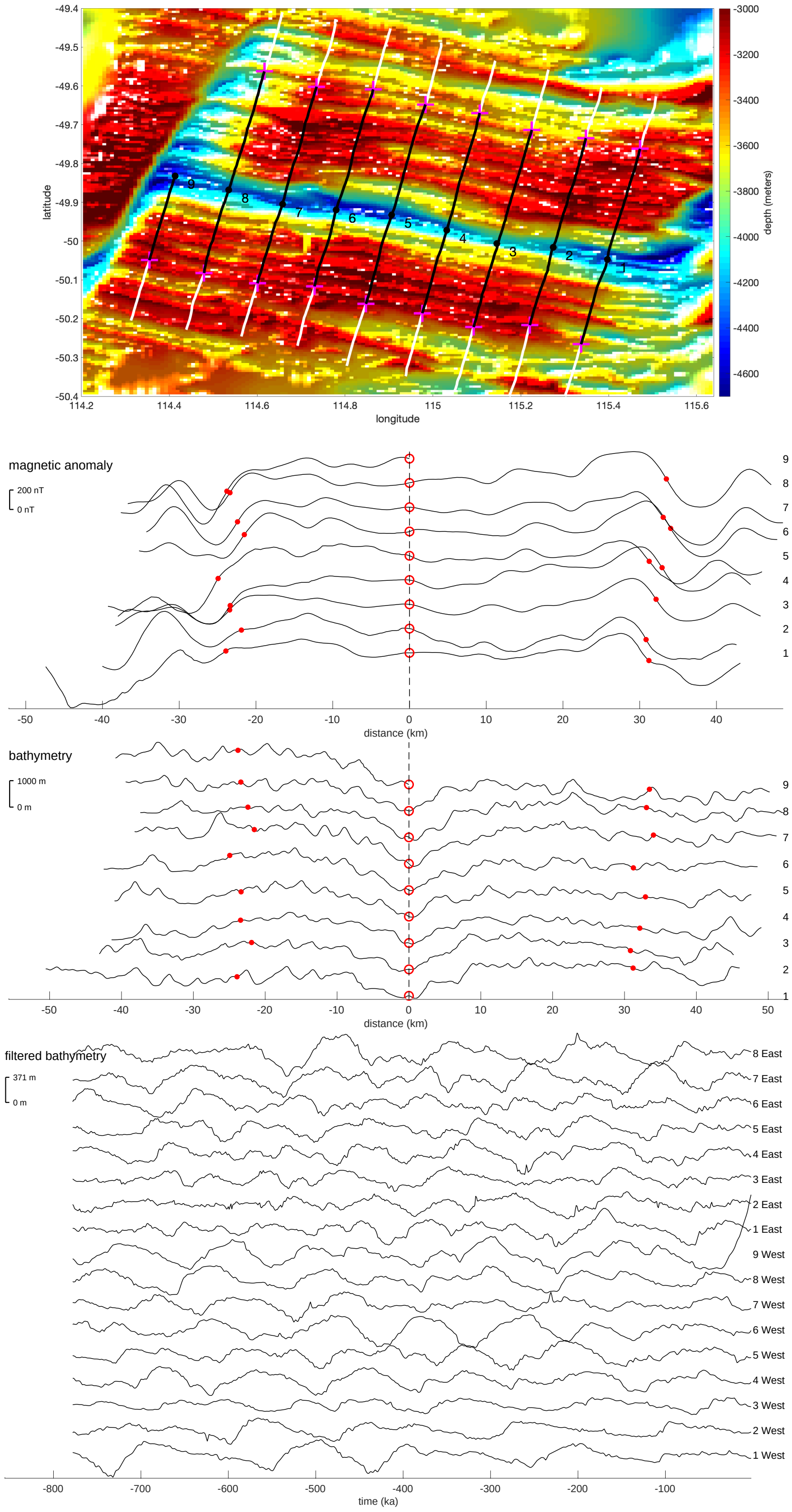

Fig. S15. Data from cruise WEST09MVa. Features as described in Fig. S7.

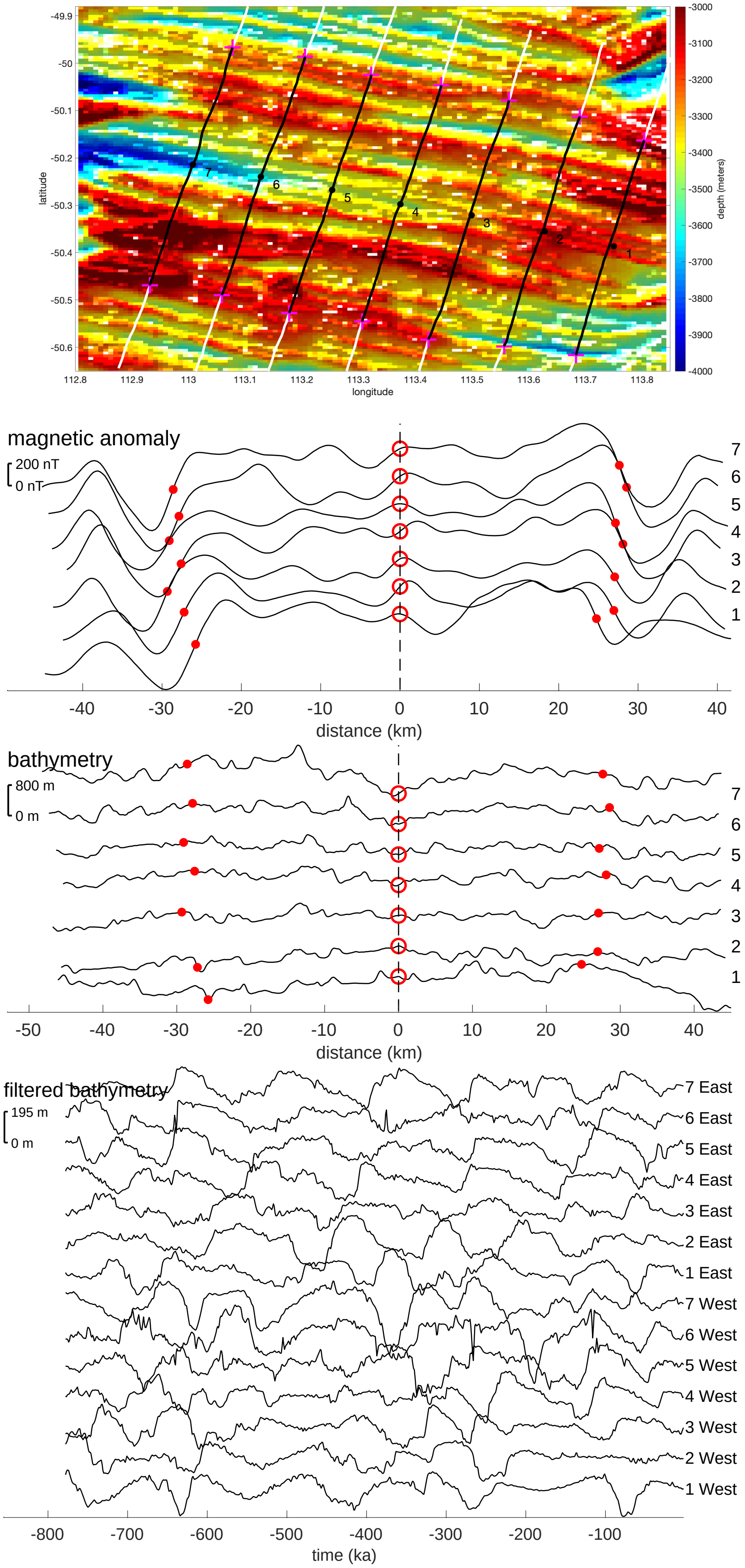

Fig. S16. Data from cruise WEST09MVb. Features as described in Fig. S7.

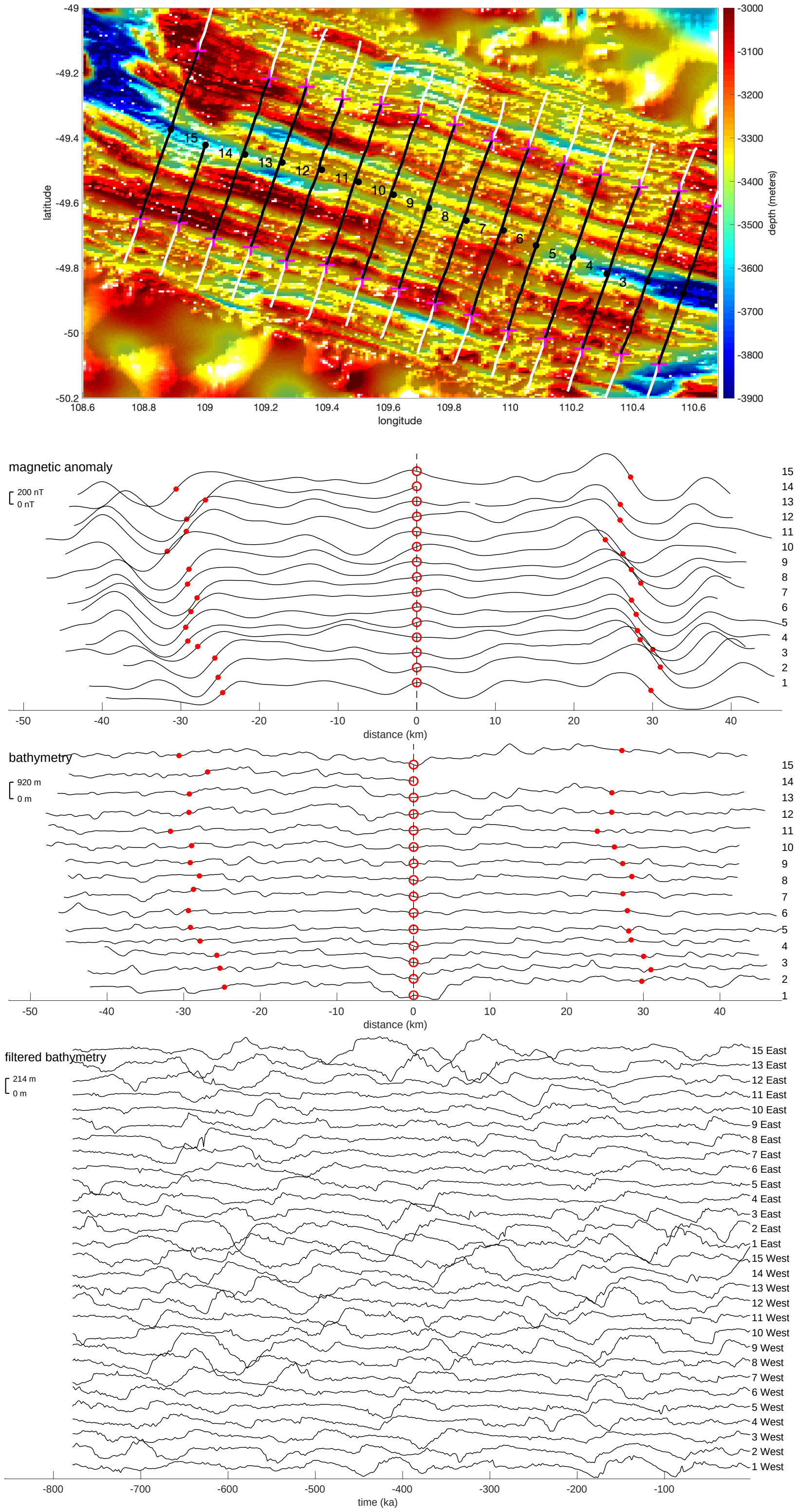

Fig. S17. Data from cruise WEST09MVc. Features as described in Fig. S7.

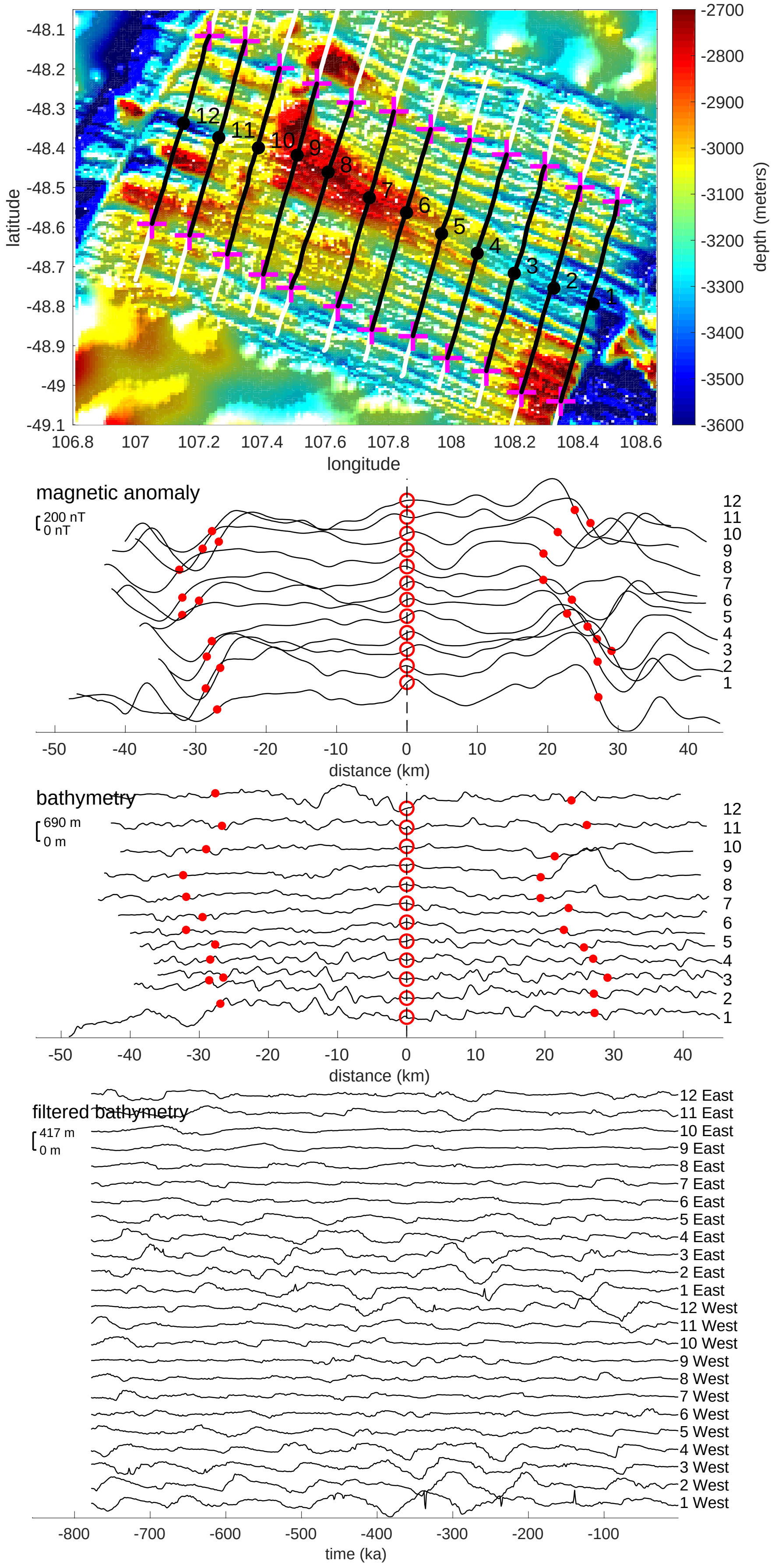

Fig. S18. Data from cruise WEST09MVd. Features as described in Fig. S7.

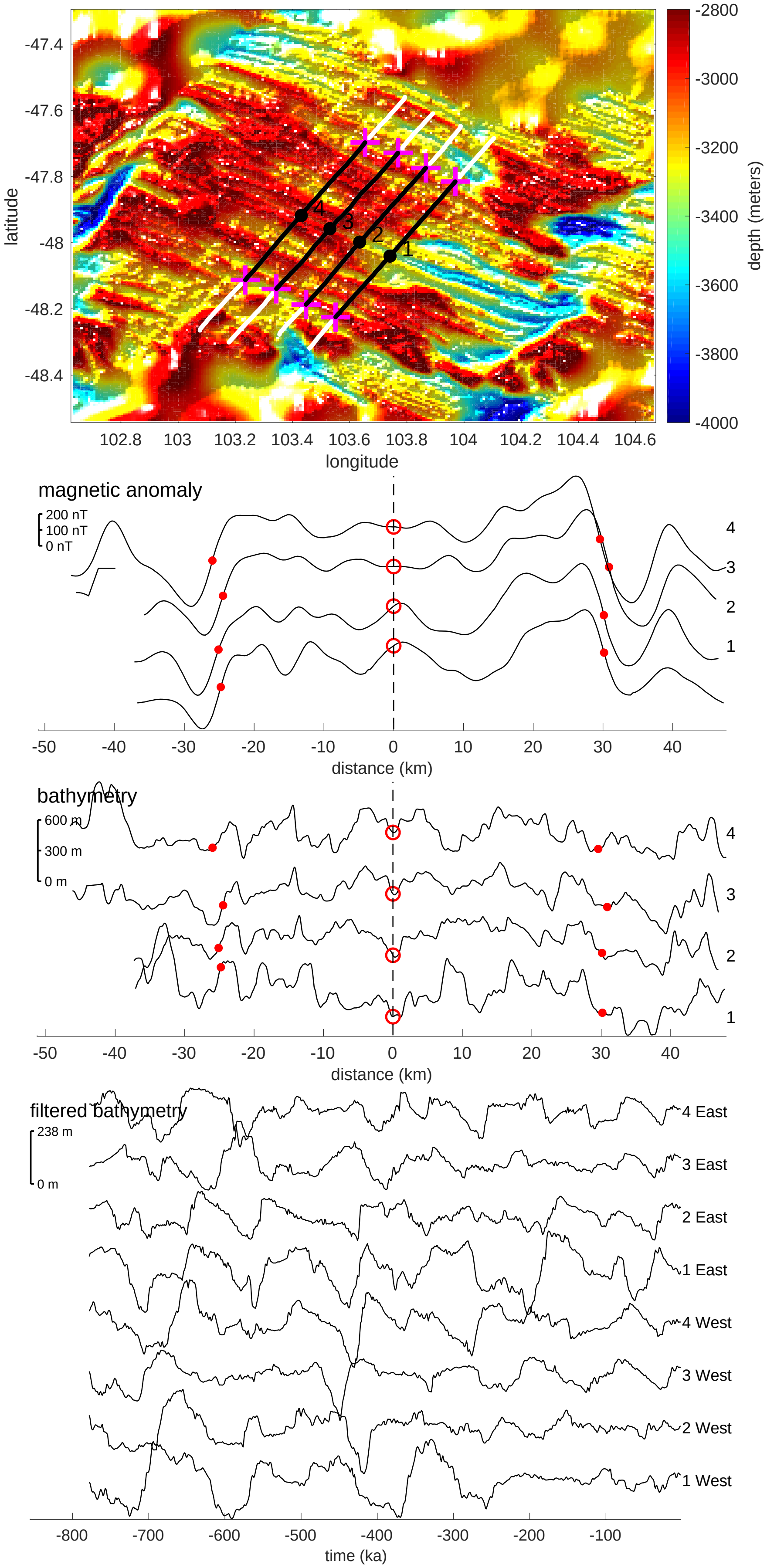

Fig. S19. Data from cruise WEST09MVe. Features as described in Fig. S7.

P. Huybers, P. Liautaud, C. Proistosescu, B. Boulahanis, S.M. Carbotte, R.F. Katz, C. Langmuir

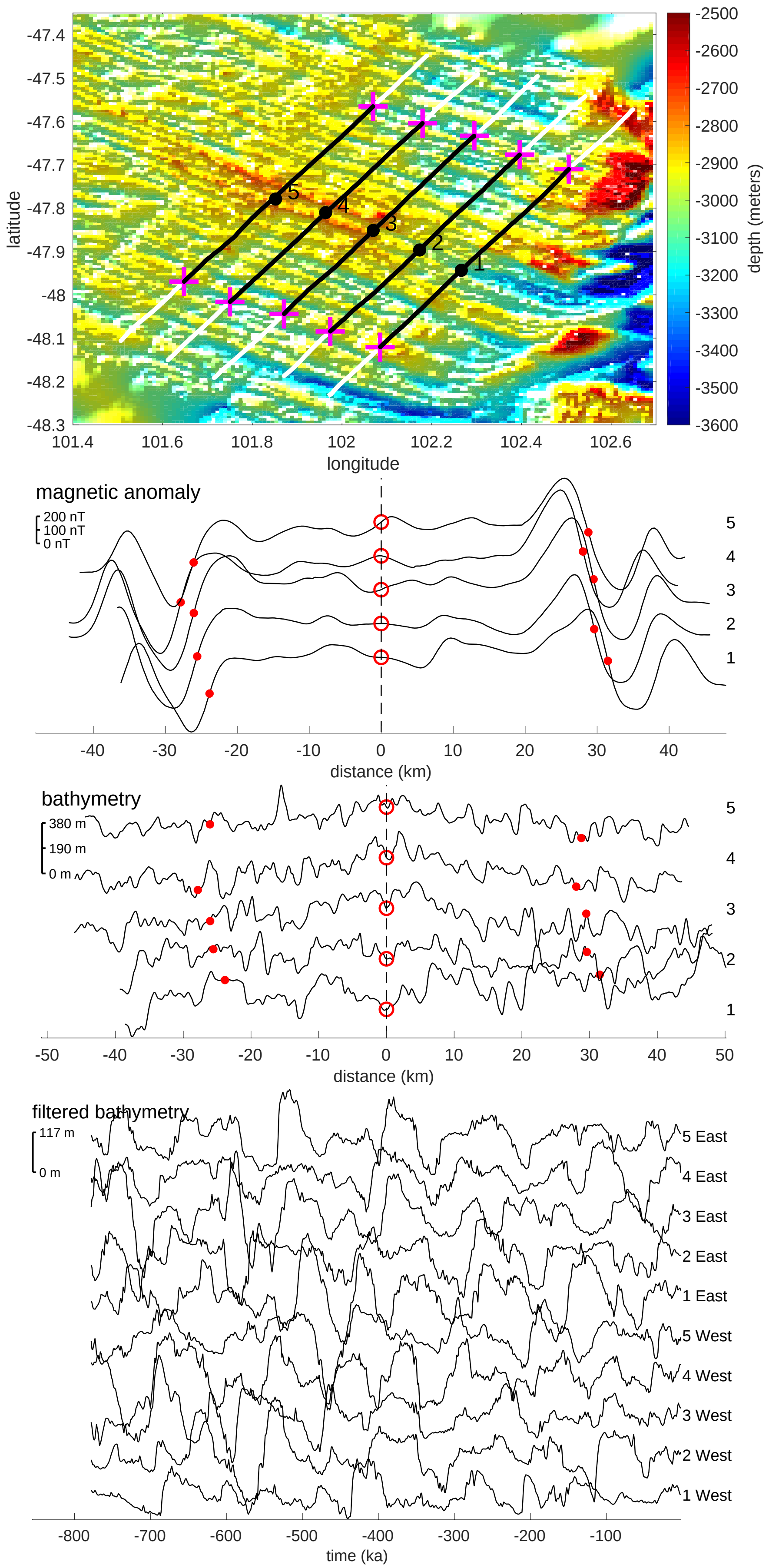

Fig. S20. Data from cruise WEST09MVf. Features as described in Fig. S7.

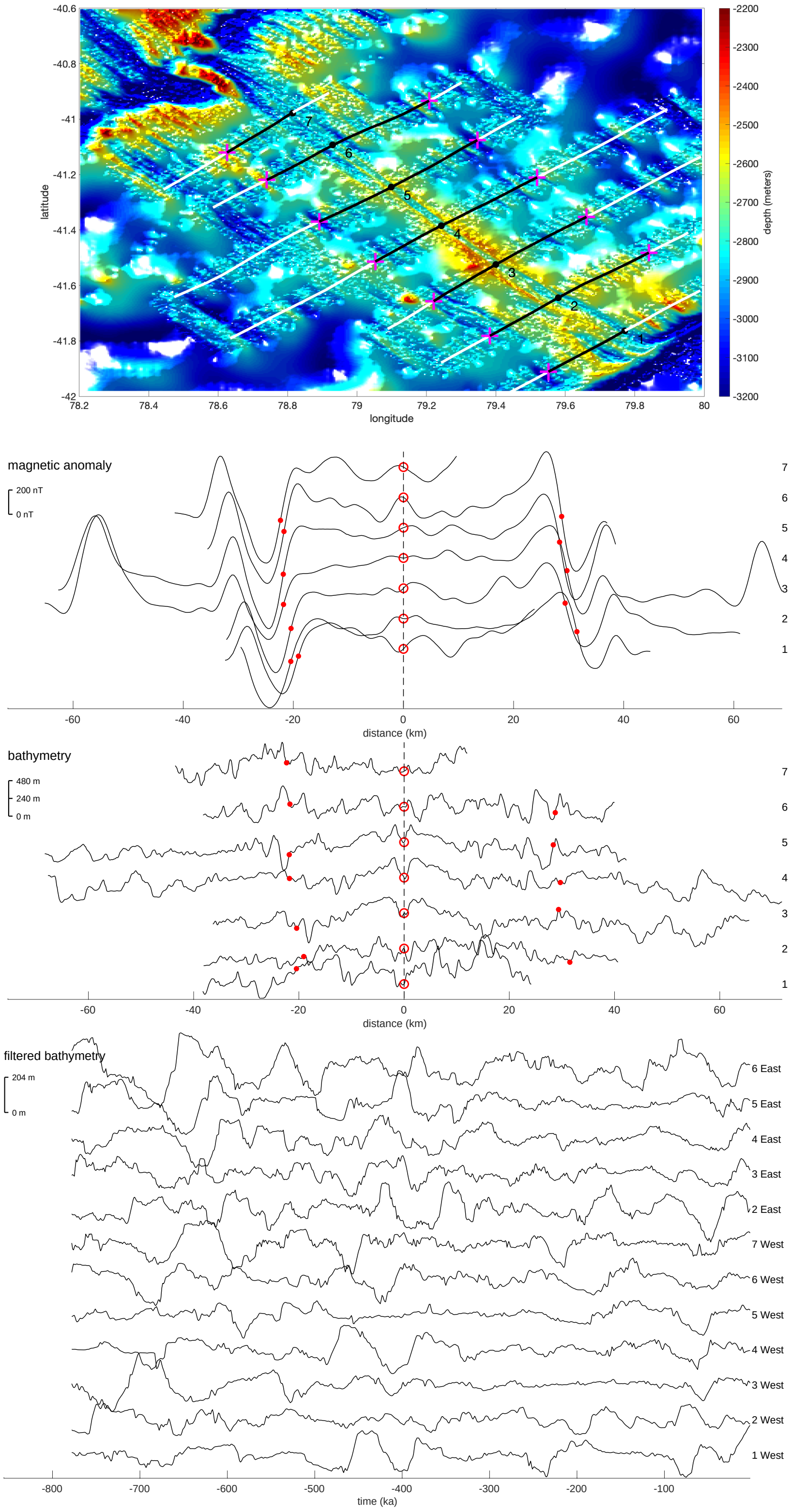

Fig. S21. Data from cruise BMRG06MV. Features as described in Fig. S7.

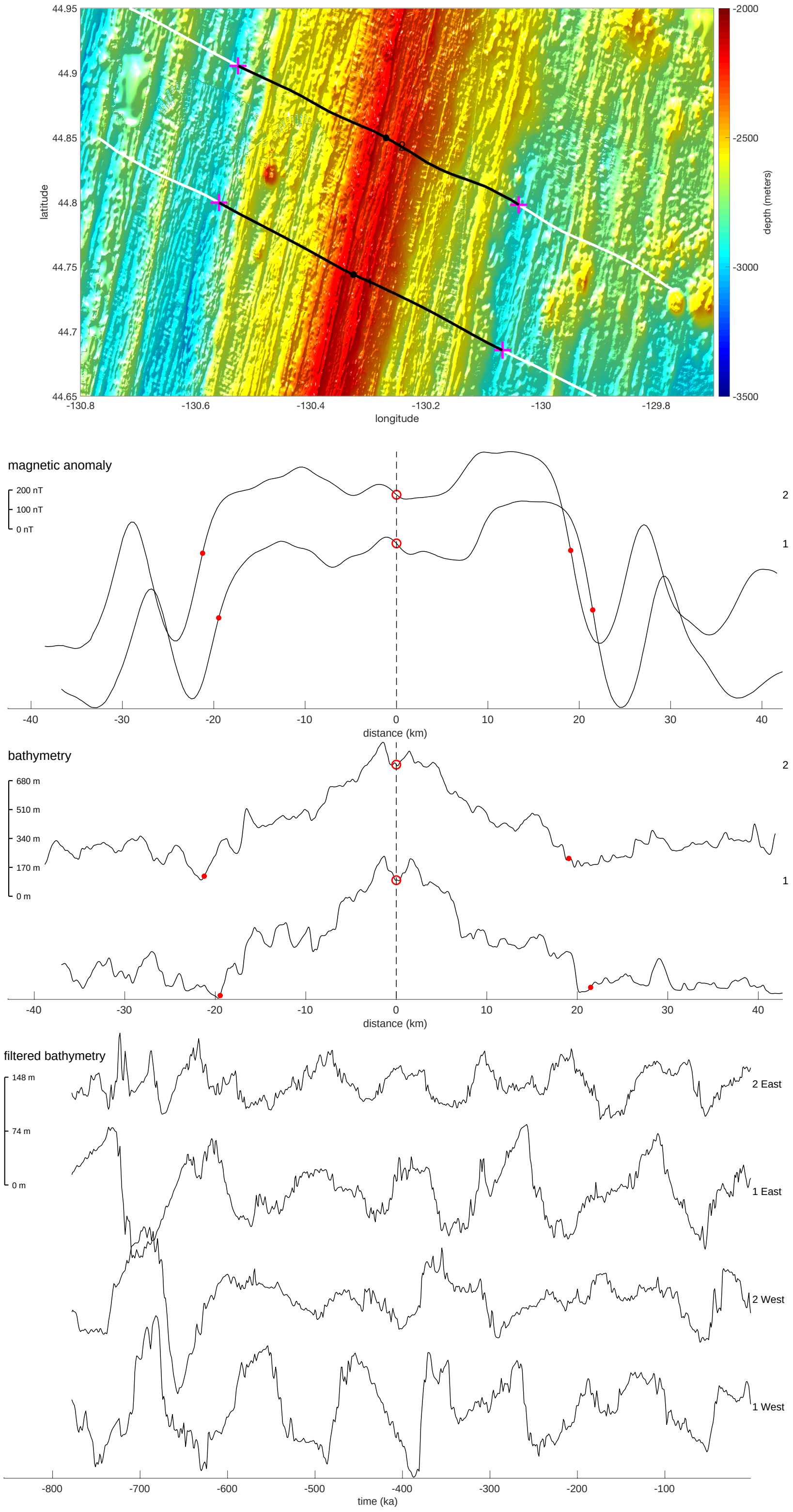

Fig. S22. Data from cruise WEST15MV. Features as described in Fig. S7.

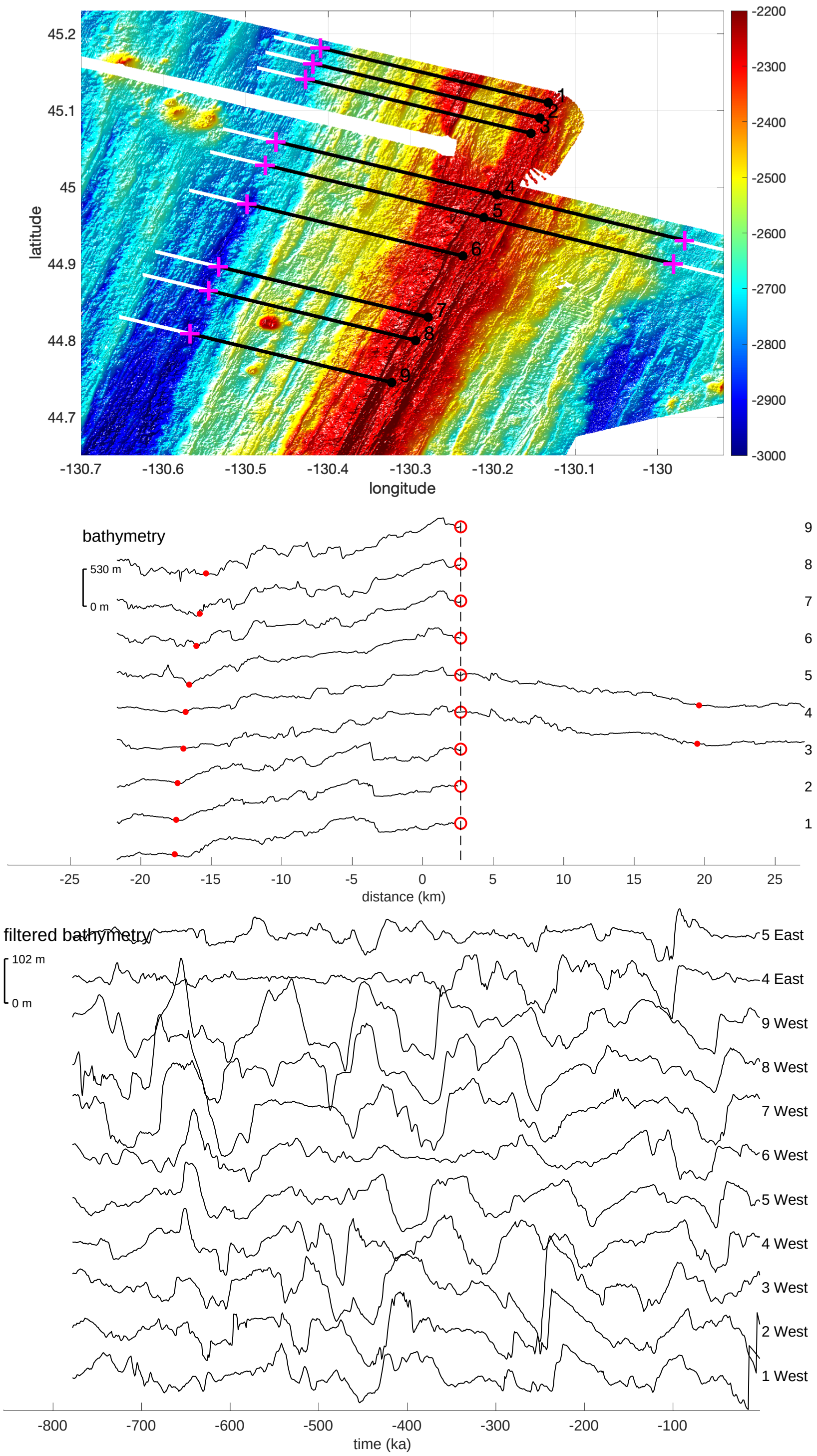

Fig. S23. Data from cruise AT26-19. Only bathymetry acquired perpendicular to the ridge axis is included in order to make results comparable with other transects and to minimize any influence from incompletely corrected ship roll. Transects are selected in order to obtain equal spacing and cover regions where data is complete. Magnetics reversals are from ref. (7). Features are otherwise as described in Fig. S7.

Table S1. Bathymetry segments analyzed in this study. Segments are grouped according to region (Figure 1 of the main text) and transect. 92 transects include segments on both sides of a ridge axis, and dashes indicate a one-sided transect. Also listed is the location of the ridge axis (°lon and °lat), average spatial resolution of the profile (m), locations of the Brunhes-Matuyama magnetic reversal (°lon and °lat), spreading half-rates (cm/yr) used in this study, and, for purposes of comparison, the estimated spreading half-rate (cm/yr) from the MORVEL plate motion model (8). The table is continued on the next page.

Reg.	Num.	Ridge	Res.	B-M 1	B-M 2	$U_0 1$ (B-M)	$U_0 2$ (B-M)	U_0 (MORVEL)
1	1	16.7395,-105.3535	67.88	16.6875,-105.6451	16.7223,-105.0609	4.043	3.929	4.123
2	1	13.1475,-104.0363	36.26	13.1675,-104.4096	13.132,-103.7031	4.968	4.444	4.666
3	1	8.6967,-104.2085	24	8.6406,-104.6578	8.7362,-103.8842	6.37	4.595	5.261
4	1	-19.855,-113.643	65.39	-19.8551,-114.1429	-19.8558,-113.0659	6.597	7.622	7.16
4	2	-19.7633,-113.6248	68.41	-19.7638,-114.1276	-19.7649,-113.0492	6.639	7.609	7.158
4	3	-19.6698,-113.6046	63.38	-19.6694,-114.1044	-19.6699,-113.0225	6.605	7.695	7.155
4	4	-19.5784,-113.5795	67.19	-19.5778,-114.086	-19.578,-113.0085	6.698	7.552	7.153
4	5	-19.4846,-113.559	56.2	-19.485,-114.0629	-19.4846,-112.9798	6.664	7.665	7.151
4	6	-19.3928,-113.5385	62.28	-19.3929,-114.0506	-19.3923,-112.9671	6.777	7.566	7.148
4	7	-19.3,-113.5109	51.66	-19.3002,-114.0191	-19.2999,-112.9412	6.729	7.549	7.146
4	8	-19.2083,-113.4954	60.61	-19.2084,-114.0046	-19.2079,-112.9185	6.747	7.647	7.143
4	9	-19.117,-113.4801	67.88	-19.117,-113.9785	-19.1161,-112.9004	6.608	7.687	7.141
5	1	-40.2002,-91.5728	77.38	-40.2002,-91.7791	-40.2006,-91.3003	2.244	2.964	2.547
5	2	-40.1167,-91.5766	66.59	-40.1162,-91.795	-40.1164,-91.3166	2.378	2.832	2.546
5	3	-40.033,-91.5831	63.98	-40.033,-91.8068	-40.0335,-91.3218	2.439	2.849	2.545
5	4	-39.9503,-91.5743	68.56	-39.95,-91.8032	-39.9498,-91.3532	2.499	2.414	2.544
5	5	-39.8675,-91.5274	71.86	-39.8658,-91.8047	-39.8662,-91.3283	3.03	2.177	2.543
5	6	-39.7834,-91.5076	65.14	-39.7831,-91.7207	-	2.333	-	2.542
5	7	-39.6992,-91.5091	64.09	-39.6998,-91.7128	-	2.233	-	2.541
5	8	-39.6167,-91.518	63.2	-39.6166,-91.7442	-	2.482	-	2.54
5	9	-39.533,-91.5309	63.41	-39.5332,-91.7542	-	2.453	-	2.539
5	10	-39.4493,-91.5425	64.12	-39.4494,-91.7634	-39.4498,-91.2797	2.43	2.89	2.538
5	11	-39.3671,-91.5527	63.43	-39.3665,-91.7615	-	2.299	-	2.537
5	12	-39.2842,-91.5606	62.26	-	-39.2841,-91.3083	-	2.781	2.536
5	13	-39.2008,-91.5652	61.47	-39.1998,-91.7847	-39.2006,-91.3084	2.422	2.834	2.535
5	14	-39.1161,-91.573	65.58	-39.117,-91.7944	-39.1161,-91.329	2.448	2.696	2.534
5	15	-39.0335,-91.5795	68.66	-39.0334,-91.7999	-39.0331,-91.3263	2.438	2.802	2.533
6	1	-49.0599,-113.5203	57.06	-48.9999,-113.9836	-49.1202,-113.0766	4.356	4.185	4.358
7	1	-64.7853,-169.9418	71.01	-64.8993,-169.6579	-64.6327,-170.3224	2.365	-	2.753
7	2	-64.76,-169.889	63.96	-64.8788,-169.5951	-64.6077,-170.2722	2.458	-	2.756
7	3	-64.7313,-169.8294	75.31	-64.8607,-169.5029	-	2.707	-	2.758
7	4	-64.7043,-169.7732	64.9	-64.8373,-169.4411	-64.5636,-170.1201	2.768	-	2.761
7	5	-64.6738,-169.7099	37.69	-64.5383,-170.0484	-64.811,-169.3709	2.83	2.84	2.764
7	6	-64.646,-169.6521	60.85	-64.5005,-169.9819	-	2.887	-	2.766
7	7	-64.4044,-171.5288	61.74	-64.2647,-171.9134	-	3.098	-	2.736
7	8	-64.3757,-171.4599	56.54	-64.2313,-171.8403	-	3.124	-	2.739
7	9	-64.346,-171.3887	66.33	-64.1999,-171.763	-64.4773,-171.083	3.114	2.65	2.742
7	10	-64.3219,-171.3307	56.05	-64.1756,-171.6902	-	3.049	-	2.744
8	1	-48.7956,127.2744	84.03	-49.0335,127.213	-48.5448,127.3407	3.44	3.63	3.508
8	2	-48.7816,127.1348	84.59	-49.03,127.0694	-48.5277,127.2021	3.594	3.675	3.509
8	3	-48.765,126.9973	87.32	-49.0077,126.934	-48.5016,127.07	3.51	3.817	3.509
8	4	-48.736,126.8625	89.37	-49.0118,126.7874	-48.4991,126.9266	3.994	3.431	3.51
8	5	-48.7211,126.7231	87.63	-48.996,126.647	-	3.984	-	3.51
8	6	-48.7002,126.5783	84.96	-48.9937,126.4984	-48.485,126.6354	4.25	3.115	3.511
8	7	-48.6767,126.4425	94.78	-48.9821,126.36	-48.4609,126.5013	4.422	3.126	3.512
9	1	-50.0475,115.3969	79.06	-50.2658,115.3372	-49.7621,115.4715	3.067	3.999	3.523
9	2	-50.0164,115.2738	65.44	-50.2165,115.2205	-49.7346,115.3482	2.807	3.952	3.523
9	3	-50.0064,115.1454	77.71	-50.2217,115.0926	-49.7133,115.225	3.004	4.119	3.523
9	4	-49.972,115.0315	74.14	-50.1858,114.9758	-49.6699,115.1065	2.995	4.22	3.523
9	5	-49.9331,114.9062	74	-50.1616,114.8476	-49.6481,114.983	3.197	4.005	3.523
9	6	-49.9194,114.7796	71.67	-50.1169,114.7304	-49.609,114.8638	2.759	4.363	3.523
9	7	-49.9049,114.6579	71.11	-50.1088,114.6004	-49.6021,114.7352	2.873	4.238	3.522
9	8	-49.8683,114.5352	76.69	-50.0822,114.4791	-49.5626,114.6165	2.998	4.291	3.522
9	9	-49.8319,114.4131	85.85	-50.0491,114.3544	-	3.05	-	3.522
10	1	-50.3863,113.7505	74.49	-50.6166,113.6836	-50.1627,113.8038	3.304	3.174	3.522
10	2	-50.3548,113.6284	73.48	-50.5978,113.5565	-50.1124,113.6905	3.489	3.455	3.521
10	3	-50.3211,113.4992	69.33	-50.5833,113.4231	-50.0785,113.5653	3.76	3.469	3.521
10	4	-50.2979,113.3735	71.79	-50.5454,113.3062	-50.0467,113.4457	3.537	3.602	3.521
10	5	-50.2675,113.2534	67.44	-50.5273,113.1775	-50.0246,113.3224	3.729	3.481	3.521
10	6	-50.2401,113.1276	71.7	-50.4898,113.0585	-49.9859,113.2055	3.573	3.66	3.52
10	7	-50.2141,113.0076	74.36	-50.4692,112.9315	-49.9668,113.0775	3.666	3.544	3.52

Reg.	Num.	Ridge	Res.	B-M 1	B-M 2	$U_0 1$ (B-M)	$U_0 2$ (B-M)	U_0 (MORVEL)
11	1	-49.8753,110.5668	63.25	-50.0957,110.485	-49.6084,110.6616	3.165	3.819	3.514
11	2	-49.8395,110.4476	64.89	-50.0646,110.3619	-49.5639,110.5525	3.24	3.971	3.513
11	3	-49.8176,110.3154	59.43	-50.0482,110.2348	-49.5518,110.4219	3.293	3.849	3.513
11	4	-49.7666,110.205	62.61	-50.0156,110.1132	-49.5121,110.2953	3.573	3.641	3.513
11	5	-49.7303,110.0842	62.42	-49.9916,109.992	-49.4796,110.1762	3.735	3.599	3.512
11	6	-49.684,109.9772	63.3	-49.9443,109.8729	-49.432,110.059	3.769	3.577	3.512
11	7	-49.6536,109.8551	60.37	-49.9079,109.7528	-49.4081,109.9391	3.683	3.502	3.511
11	8	-49.6156,109.7329	60.84	-49.8634,109.6348	-49.3587,109.8176	3.584	3.652	3.511
11	9	-49.5745,109.6178	61.98	-49.8328,109.515	-49.3283,109.6988	3.738	3.499	3.51
11	10	-49.5349,109.5027	60.59	-49.7906,109.3972	-49.2972,109.5764	3.715	3.361	3.51
11	11	-49.4983,109.383	64.92	-49.7781,109.2667	-49.281,109.4508	4.069	3.074	3.509
11	12	-49.4754,109.2538	64.32	-49.735,109.1502	-49.2425,109.3315	3.759	3.315	3.509
11	13	-49.451,109.1316	64.9	-49.71,109.028	-49.2181,109.2101	3.752	3.318	3.508
11	14	-49.4215,109.0018	61.69	-49.6618,108.9144	-	3.447	-	3.508
11	15	-49.3747,108.8878	66.58	-49.6482,108.7876	-49.1322,108.9773	3.925	3.486	3.507
12	1	-48.7947,108.4477	58.64	-49.0405,108.3448	-48.5353,108.5237	3.457	3.484	3.504
12	2	-48.7544,108.3229	63.85	-49.0176,108.2201	-48.4993,108.406	3.664	3.471	3.503
12	3	-48.7169,108.1975	55.24	-48.9642,108.1091	-48.4464,108.2942	3.401	3.725	3.503
12	4	-48.6659,108.08	65.56	-48.9311,107.9858	-48.4163,108.1734	3.645	3.458	3.502
12	5	-48.6169,107.9672	59.79	-48.8759,107.8767	-48.3798,108.0562	3.552	3.288	3.501
12	6	-48.5635,107.8563	63.98	-48.8595,107.7466	-48.3523,107.9331	4.092	2.915	3.501
12	7	-48.5257,107.7387	65.1	-48.8002,107.6388	-48.3075,107.8162	3.787	3.002	3.5
12	8	-48.461,107.6082	60.73	-48.7535,107.4911	-48.2849,107.6819	4.09	2.482	3.499
12	9	-48.4183,107.51	62.91	-48.7199,107.4029	-48.2371,107.5726	4.147	2.485	3.499
12	10	-48.3995,107.3875	60.7	-48.669,107.2892	-48.1984,107.4545	3.72	2.746	3.498
12	11	-48.3736,107.2618	65.26	-48.621,107.1693	-48.1299,107.3456	3.427	3.341	3.498
12	12	-48.3373,107.1502	58.37	-48.5918,107.0504	-48.1167,107.232	3.55	3.055	3.497
13	1	-48.0405,103.7411	67.3	-48.225,103.5506	-47.8151,103.9698	3.178	3.867	3.478
13	2	-47.9981,103.635	58.19	-48.187,103.4475	-47.7742,103.8667	3.219	3.862	3.477
13	3	-47.9575,103.5312	65.34	-48.1396,103.3433	-47.7284,103.7693	3.137	3.957	3.476
13	4	-47.9182,103.4306	56.44	-48.1129,103.2349	-47.6971,103.6541	3.331	3.79	3.475
14	1	-47.9435,102.2659	63.27	-48.1207,102.0849	-47.7101,102.5056	3.058	4.038	3.468
14	2	-47.8967,102.173	55.13	-48.0843,101.9736	-47.6767,102.3962	3.277	3.794	3.468
14	3	-47.8515,102.0694	64.85	-48.0443,101.8706	-47.6334,102.2948	3.338	3.783	3.467
14	4	-47.8104,101.9635	52.32	-48.0163,101.75	-47.6041,102.1796	3.571	3.593	3.466
14	5	-47.7792,101.852	61.56	-47.9698,101.6476	-47.5655,102.0692	3.341	3.69	3.465
15	1	-41.7634,79.7712	56	-41.9114,79.5513	-	2.623	-	3.155
15	2	-41.6445,79.5795	60.13	-41.7818,79.3828	-41.4817,79.8406	2.445	4.039	3.15
15	3	-41.5247,79.3999	56.49	-41.657,79.2206	-41.3528,79.6609	2.618	3.764	3.145
15	4	-41.385,79.2426	58.31	-41.5138,79.0526	-41.2102,79.5187	2.793	3.808	3.14
15	5	-41.245,79.099	58.86	-41.3698,78.8912	-41.0757,79.3466	2.798	3.635	3.135
15	6	-41.0932,78.9297	56.06	-41.2192,78.7402	-40.9337,79.2077	2.782	3.683	3.129
15	7	-40.9784,78.8151	45.92	-41.1205,78.6255	-	2.864	-	3.125
16	1	44.7441,-130.3258	47.44	44.7998,-130.5595	44.6856,-130.0667	2.495	2.753	2.476
16	2	44.8498,-130.269	44.72	44.9055,-130.5266	44.7981,-130.0385	2.528	2.729	2.481
17	1	45.1814,-130.4096	29.64	45.11,-130.1328	-	2.853	-	2.493
17	2	45.161,-130.4185	29.49	45.09,-130.1432	-	2.838	-	2.492
17	3	45.1407,-130.4274	29.34	45.07,-130.1536	-	2.824	-	2.491
17	4	45.0592,-130.4631	28.74	44.99,-130.1952	44.9304,-129.9672	2.766	2.36	2.488
17	5	45.0287,-130.4764	28.51	44.96,-130.2108	44.9,-129.9811	2.744	2.378	2.486
17	6	44.9778,-130.4987	28.13	44.91,-130.2368	-	2.708	-	2.484
17	7	44.9304,-129.9672	24.52	44.99,-130.1952	-	2.36	-	2.487
17	8	44.9,-129.9811	24.71	44.96,-130.2108	-	2.378	-	2.486
17	9	44.896,-130.533	27.4	44.83,-130.2784	-	2.637	-	2.48

Table S2. **Inferred significance of the 1/(41 ky) spectral peak under 72 different formulations of spectral analysis. Formulations consist of different time-bandwidth products (t.b.), autoregressive orders (AR), moving average orders (MA), interpolation spacing (dt in ky), and whether time-series are differenced (diff., 1 is yes and 0 is no). Also given are the p-values for the 1/(100 ky) band at intermediate spreading ridges ($\geq$2.3 and $<$3.8 cm/yr), and the p-values for fast-spreading ridges ($\geq$4 cm/yr). The main manuscript shows specification 71 (see Fig. S5). Also given are the expected variance of the logarithm of the spectrum (exp.) and the observed variance (obs.).**

form	t.b.	AR	MA	dt	diff.	p-value	exp.	obs.
1	3	1	0	1	0	0	0.221	1.220
2	3	1	0	1	1	0	0.221	0.531
3	3	1	0	5	0	0	0.221	0.604
4	3	1	0	5	1	0	0.221	0.368
5	3	1	1	1	0	0	0.221	0.405
6	3	1	1	1	1	0	0.221	0.294
7	3	1	1	5	0	0	0.221	0.263
8	3	1	1	5	1	0	0.221	0.305
9	3	1	2	1	0	0	0.221	0.328
10	3	1	2	1	1	0	0.221	0.278
11	3	1	2	5	0	0.009	0.221	0.205
12	3	1	2	5	1	0	0.221	0.197
13	3	2	0	1	0	0	0.221	0.517
14	3	2	0	1	1	0	0.221	0.414
15	3	2	0	5	0	0	0.221	0.240
16	3	2	0	5	1	0	0.221	0.262
17	3	2	1	1	0	0	0.221	0.279
18	3	2	1	1	1	0	0.221	0.286
19	3	2	1	5	0	0.001	0.221	0.199
20	3	2	1	5	1	0.039	0.221	0.205
21	3	2	2	1	0	0	0.221	0.515
22	3	2	2	1	1	0	0.221	0.266
23	3	2	2	5	0	0.006	0.221	0.179
24	3	2	2	5	1	0.025	0.221	0.176
25	4	1	0	1	0	0	0.154	1.135
26	4	1	0	1	1	0	0.154	0.453
27	4	1	0	5	0	0	0.154	0.530
28	4	1	0	5	1	0	0.154	0.282
29	4	1	1	1	0	0	0.154	0.330
30	4	1	1	1	1	0	0.154	0.218
31	4	1	1	5	0	0	0.154	0.192
32	4	1	1	5	1	0	0.154	0.222
33	4	1	2	1	0	0	0.154	0.255
34	4	1	2	1	1	0	0.154	0.201
35	4	1	2	5	0	0.003	0.154	0.135
36	4	1	2	5	1	0	0.154	0.124
37	4	2	0	1	0	0	0.154	0.441
38	4	2	0	1	1	0	0.154	0.337
39	4	2	0	5	0	0	0.154	0.169
40	4	2	0	5	1	0	0.154	0.181
41	4	2	1	1	0	0	0.154	0.205
42	4	2	1	1	1	0	0.154	0.210
43	4	2	1	5	0	0	0.154	0.129
44	4	2	1	5	1	0.023	0.154	0.130
45	4	2	2	1	0	0	0.154	0.437
46	4	2	2	1	1	0	0.154	0.189
47	4	2	2	5	0	0.002	0.154	0.112
48	4	2	2	5	1	0.012	0.154	0.103
49	5	1	0	1	0	0	0.118	1.093

13

50	5	1	0	1	1	0	0.118	0.411
51	5	1	0	5	0	0	0.118	0.492
52	5	1	0	5	1	0	0.118	0.230
53	5	1	1	1	0	0	0.118	0.292
54	5	1	1	1	1	0	0.118	0.177
55	5	1	1	5	0	0	0.118	0.153
56	5	1	1	5	1	0	0.118	0.172
57	5	1	2	1	0	0	0.118	0.216
58	5	1	2	1	1	0	0.118	0.160
59	5	1	2	5	0	0.007	0.118	0.097
60	5	1	2	5	1	0	0.118	0.087
61	5	2	0	1	0	0	0.118	0.401
62	5	2	0	1	1	0	0.118	0.296
63	5	2	0	5	0	0	0.118	0.130
64	5	2	0	5	1	0	0.118	0.133
65	5	2	1	1	0	0	0.118	0.165
66	5	2	1	1	1	0	0.118	0.169
67	5	2	1	5	0	0.001	0.118	0.092
68	5	2	1	5	1	0.052	0.118	0.090
69	5	2	2	1	0	0	0.118	0.398
70	5	2	2	1	1	0	0.118	0.148
71	5	2	2	5	0	0.004	0.118	0.076
72	5	2	2	5	1	0.021	0.118	0.068
form	t.b.	AR	MA	dt	diff.	p-value	exp.	obs.

References

1. M Siddall, B Hoenisch, C Waelbroeck, P Huybers, Changes in deep Pacific temperature during the mid-Pleistocene transition and Quaternary. *Quat. Sci. Rev.* (2010).
2. JW Crowley, RF Katz, P Huybers, CH Langmuir, SH Park, Glacial cycles drive variations in the production of oceanic crust. *Science* **347**, 1237–1240 (2015).
3. JP Morgan, YJ Ghen, Dependence of ridge-axis morphology on magma supply and spreading rate. *Nature* **364**, 706 (1993).
4. GE Box, GM Jenkins, GC Reinsel, GM Ljung, *Time series analysis: forecasting and control.* (John Wiley & Sons), (2015).
5. P Huybers, Putting the significance of spectral peaks on the level: implications for the 1470-yr peak in Greenland δ^{18}O. *J. Clim.* (in review).
6. M Tolstoy, Mid-ocean ridge eruptions as a climate valve. *Geophys. Res. Lett.* **42**, 1346–1351 (2015).
7. DS Wilson, S Kirby, K Wang, S Dunlop, The Juan de Fuca plate and slab: Isochron structure and Cenozoic plate motions. *The Cascadia Subduction Zone Relat. Subduction Syst.* **4350**, 9–12 (2002).
8. C DeMets, RG Gordon, DF Argus, Geologically current plate motions. *Geophys. J. Int.* **181**, 1–80 (2010).
